# Supplementary material for: Engineering Pyrrolysyl-tRNA Synthetase for the Incorporation of Non-Canonical Amino Acids with Smaller Side Chains
Source: Int J Mol Sci. 2021 Oct 17;22(20):11194. doi: 10.3390/ijms222011194 (PMC8538471; doi:10.3390/ijms222011194)
Supplement: Supplementary file 1 [file ijms-22-11194-s001.zip › ijms-1407960-supplementary.pdf]

Supporting Information

Engineering Pyrrolysyl-tRNA Synthetase for the Incorporation of Non-Canonical Amino Acids with Smaller Side Chains

Nikolaj G. Koch[1,2], Peter Goettig[3], Juri Rappsilber[2,4] and Nediljko Budisa[1,5,*]

[1] Institut für Chemie, Technische Universität Berlin, 10623 Berlin, Germany; nikolaj.koch@mail.tu-berlin.de [2] Institut für Biotechnologie-Bioanalytik, Technische Universität Berlin, 10623 Berlin, Germany. juri.rappsilber@tu-berlin.de

[3] Structural Biology Group, Department of Biosciences, University of Salzburg, 5020 Salzburg, Austria; peter.goettig@plus.ac.at

[4] Wellcome Centre for Cell Biology, University of Edinburgh, Edinburgh EH9 3BF, United Kingdom.

[5] Department of Chemistry, University of Manitoba, Winnipeg, MB R3T 2N2, Canada.

* Correspondence: nediljko.budisa@tu-berlin.de; nediljko.budisa@umanitoba.ca; Tel.: +49-30-314-28821

Table of Contents

1. Supplementary Table 2

2. Supplementary Results 2

2.1. Previously published N-terminal PylRS mutations 2

2.2. Intact Cell Fluorescence Assays 3

2.3. Mass Spectrometry Data 11

2.4. DNA sequences used in this study 25

References: 26

1. Supplementary Table

Table S1. Amino acids used in this study.

No.	Name	Cas No.	Company
**1**	*S*-allyl-L-cystein	21593-77-1	TCI Deutschland
**2**	(*S*)-2-aminoheptanoic acid	44902-02-5	Fluorochem
**3**	(*S*)-2-aminooctanoic acid	116783-26-7	Fluorochem
**4**	(*S*)-2-aminohept-6-enoic acid	166734-64-1	Chempur
**5**	(*S*)-2-aminohexanoic acid	327-57-1	TCI Deutschland
**6**	(*S*)-2-aminohex-5-enoic acid	90989-12-1	Fluorochem
**7**	(*S*)-2-aminopentanoic acid	6600-40-4	TCI Deutschland
**8**	(*S*)-2-aminopent-4-enoic acid	16338-48-0	Fluorochem
**9**	(*S*)-2-amino-3-cyclopropylpropanoic acid	102735-53-5	Fluorochem
**10**	(*S*)-2-aminobutyric acid	1492-24-6	TCI Deutschland
**11**	(*S*)-2-aminohept-6-ynoic acid	835627-45-7	Chiralix
**12**	(*S*)-2-aminohex-5-ynoic acid	98891-36-2	Toronto Research Chemicals
**13**	(*S*)-2-aminopent-4-ynoic acid	23235-01-0	Fluorochem
**14**	(*S*)-2-amino-3-azidopropanoic acid hydrochloride	105661-40-3	Iris Biotech
**15**	(*S*)-2-amino-4-azidobutanoic acid hydrochloride	942518-29-8	Carl Roth
**16**	(*S*)-2-amino-5-azidopentanoic acid hydrochloride	1782935-10-7	Iris Biotech
**17**	(*S*)-2-amino-6-azidohexanoic acid hydrochloride	159610-92-1	Fluorochem
**18**	(*S*)-2-amino-3-cyanopropanoic acid	6232-19-5	Iris Biotech
**19**	(*S*)-2-amino-4-cyanobutanoic acid	6232-22-0	Iris Biotech
**20**	(*S*)-2-amino-5,5'-azi-hexanoic acid	851960-68-4	Thermo Fisher Scientific
**21**	(*S*)-2-amino-4-methylpent-4-enoic acid	87392-13-0	Fluorochem
**22**	L-methionine	63-68-3	Carl Roth
**23**	L-methionine sulfoxide	3226-65-1	Sigma-Aldrich (Merck)
**24**	L-methionine sulfone	7314-32-1	Iris biotech
**25**	L-ethionine	13073-35-3	Sigma-Aldrich (Merck)
**26**	*S*-tert-butyl- L-cystein	2481-09-6	Fluorochem
**27**	*S*-propargyl- L-cystein	3262-64-4	Fluorochem
**28**	*S*-benzyl- L-cystein	3054-01-1	Fluorochem

2. Supplementary Results

2.1. Previously published N-terminal PylRS mutations

In an earlier study, seven N-terminal mutations were shown to increase the ribosomal *N*εcrotonyl-lysine incorporation efficiency of an OTS based on an engineered *Mb*PylRS enzyme [62].

It was claimed that these mutations could generally increase ncAA incorporation efficiencies for PylRSbased systems, as they do not alter the aaRS substrate specificity towards ncAAs. Herein, four of these mutations were tested. Transferred to the OTS for ribosomal Sac incorporation, mutations T13I and I36V resulted in a modest enhancement of reporter production in comparison to *Mb*SacRS lacking these mutations (**Figure S1**). Based on these results, the two mutations were incorporated by default in all *Mb*SacRS constructs. All tested PylRS variants carry the Y384F mutation by default (see main text).

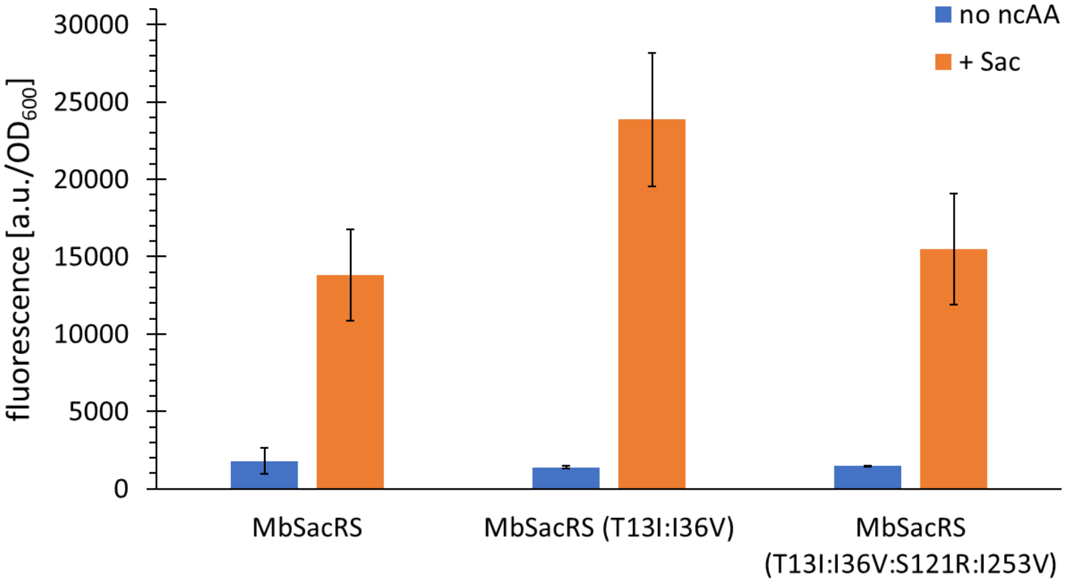

Supplementary Figure S1: OTS efficiency comparison of three different *Mb*SacRS constructs. Ribosomal incorporation of Sac (1 mM) and controls without ncAA (- Sac) supplementation. Intact cell fluorescence of *E. coli* BL21(DE3), with endpoint measurements after 24 h of incubation. Relative fluorescence is normalized to the highest value. The data (incl. standard deviation) represent the mean of three biological replicates.

2.2. Intact Cell Fluorescence Assays

Note that all following *Mb*Pyl constructs are N-terminally fused to the C-terminus of SmbP. For clarity SmbP is omitted in the notation.

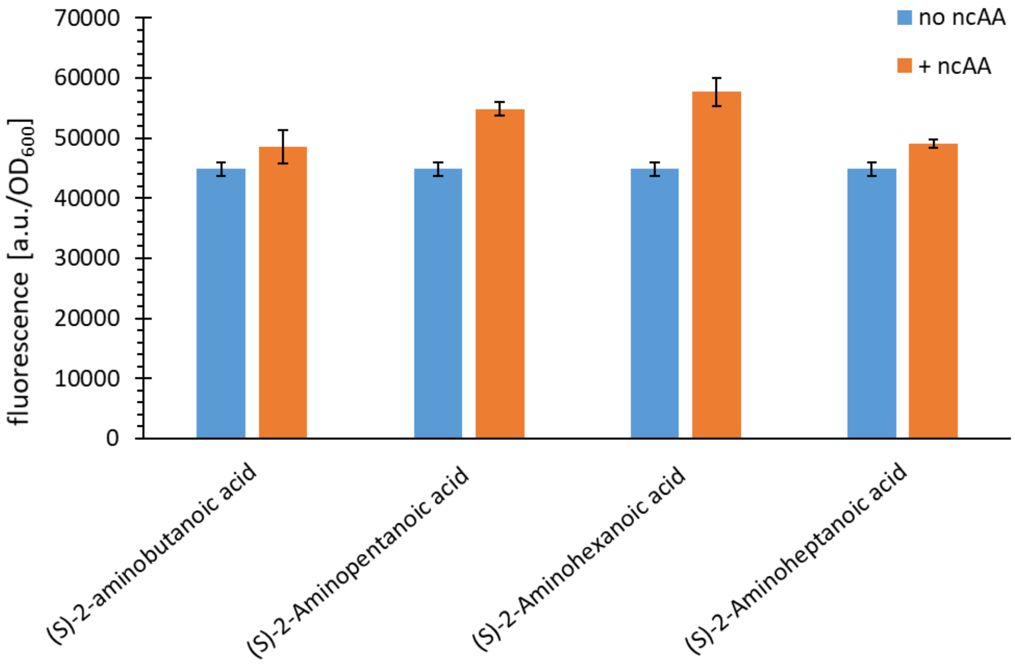

Supplementary Figure S2: Comparison of ribosomal incorporation efficiency of *Mb*SacRS with four different ncAAs (**2**, **3**, **5**, **7**, **10**) (10 mM) and controls without ncAA supplementation. Intact cell fluorescence of *E. coli* BL21(DE3), endpoint measurements after 24 h of incubation. The data (incl. standard deviation) represent the mean of three biological replicates.

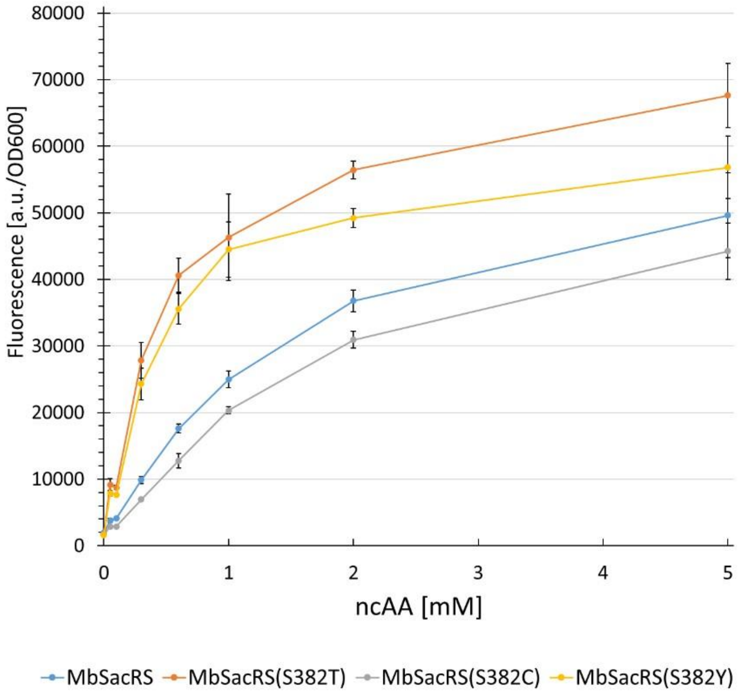

Supplementary Figure S3: Concentration dependent protein production for *Mb*PylRS variants. The measured fluorescence intensity is displayed for intact *E. coli* BL21(DE3) cells expressing the sfGFP(R2 amber) reporter, with endpoint measurements after 24 h. Endpoint measurements comprise different ncAA concentrations (0.05, 0.1, 0.3, 0.6, 1, 2, and 5 mM) of *S*-allyl-L-cysteine (Sac, **1**). The data (incl. standard deviation) represent the mean of three biological replicates.

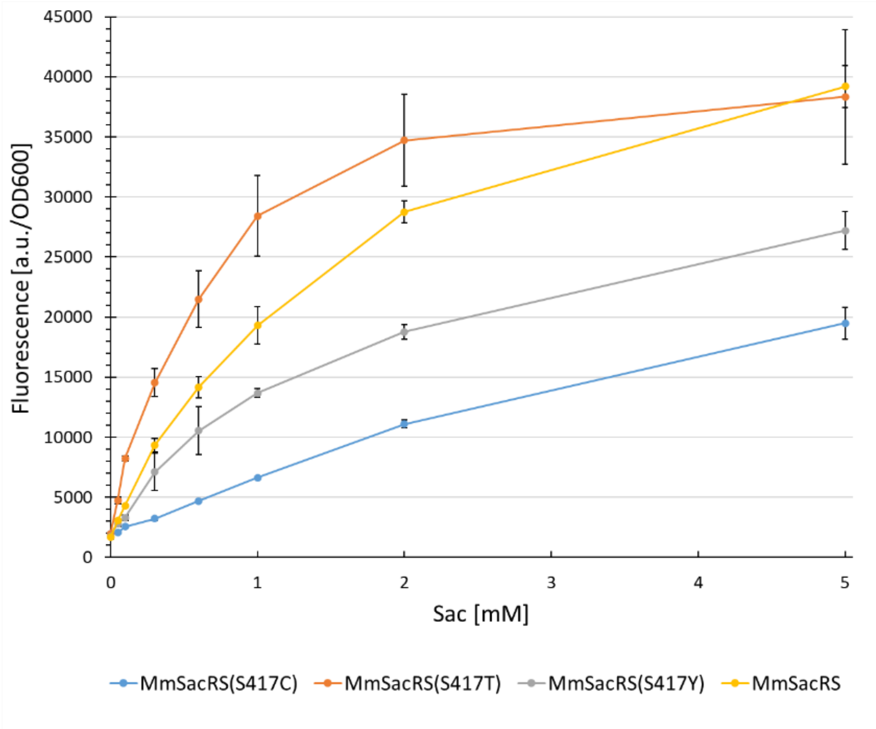

Supplementary Figure S4: Concentration dependent protein production for *Mm*PylRS variants. Measured fluorescence intensity of intact *E. coli* BL21(DE3) cells expressing the sfGFP(R2 amber) reporter, with endpoint measurements after 24 h. The endpoint measurements comprise different ncAA concentrations (0.05, 0.1, 0.3, 0.6, 1, 2, and 5 mM) of *S*-allyl-L-cysteine (Sac, **1**). The data (incl. standard deviation) represent the mean of three biological replicates.

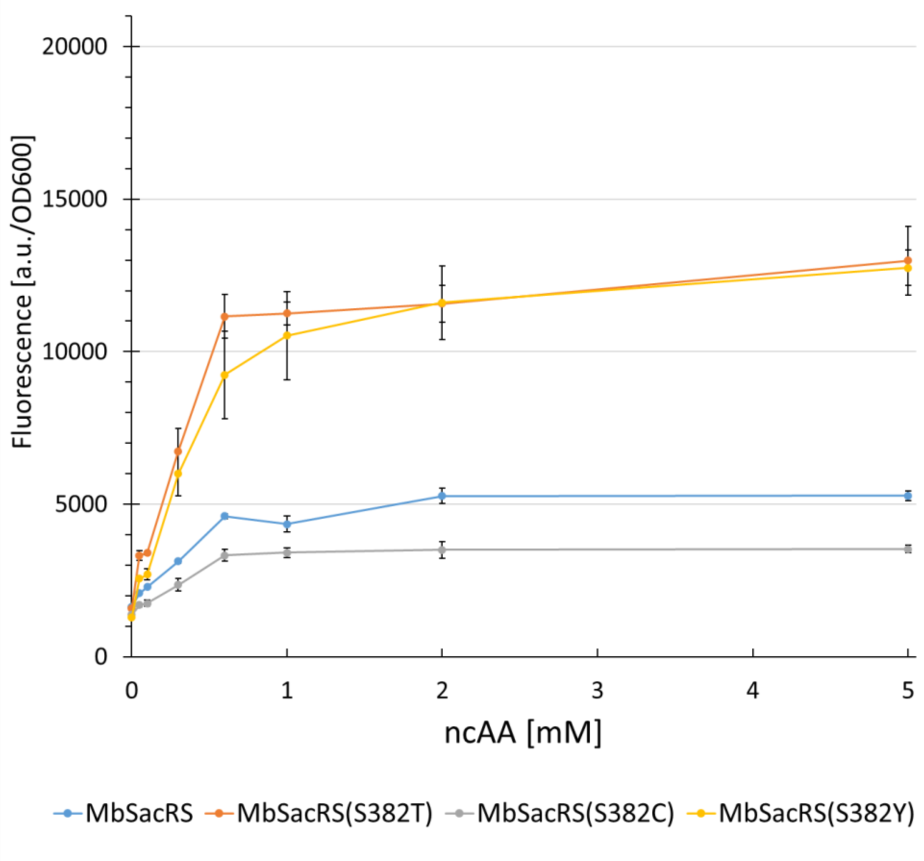

Supplementary Figure S5: Concentration dependent protein production for *Mb*PylRS variants. Measured fluorescence intensity of intact *E. coli* BL21(DE3) cells expressing the sfGFP(R2 amber)

reporter, with endpoint measurements after 24 h. Endpoint measurements comprise different ncAA concentrations (0.05, 0.1, 0.3, 0.6, 1, 2, and 5 mM) of *S*-propargyl-L-cysteine (**27**). The data (incl. standard deviation) represent the mean of three biological replicates.

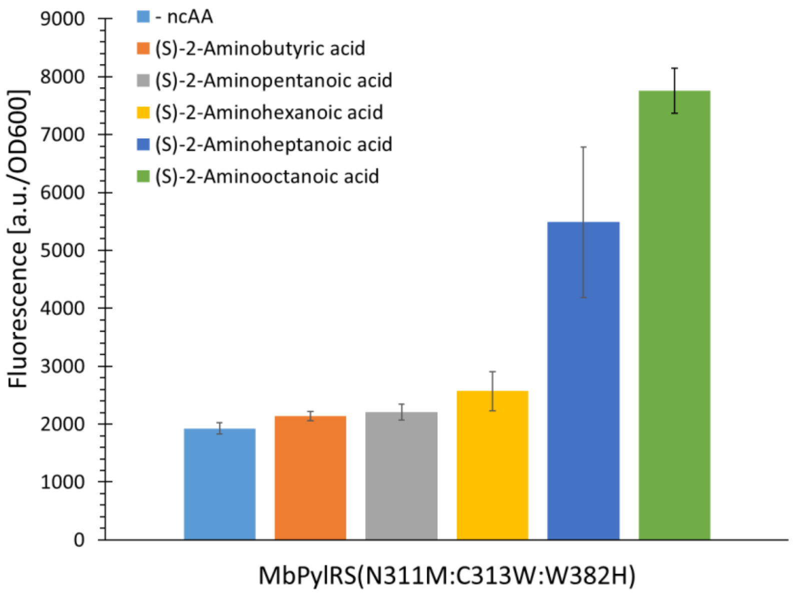

Supplementary Figure S6: Comparison of aliphatic ncAA (**2**, **3**, **5**, **7**, **10)** incorporation efficiency for *Mb*PylRS(N311M:C313W:W382H). Fluorescence measurements of intact *E. coli* BL21(DE3) cells producing the SUMO-sfGFP(R2amber) reporter protein. Intact cell fluorescence of *E. coli* BL21(DE3), with endpoint measurements after 24 h of incubation. The data (incl. standard deviation) represent the mean of three biological replicates.

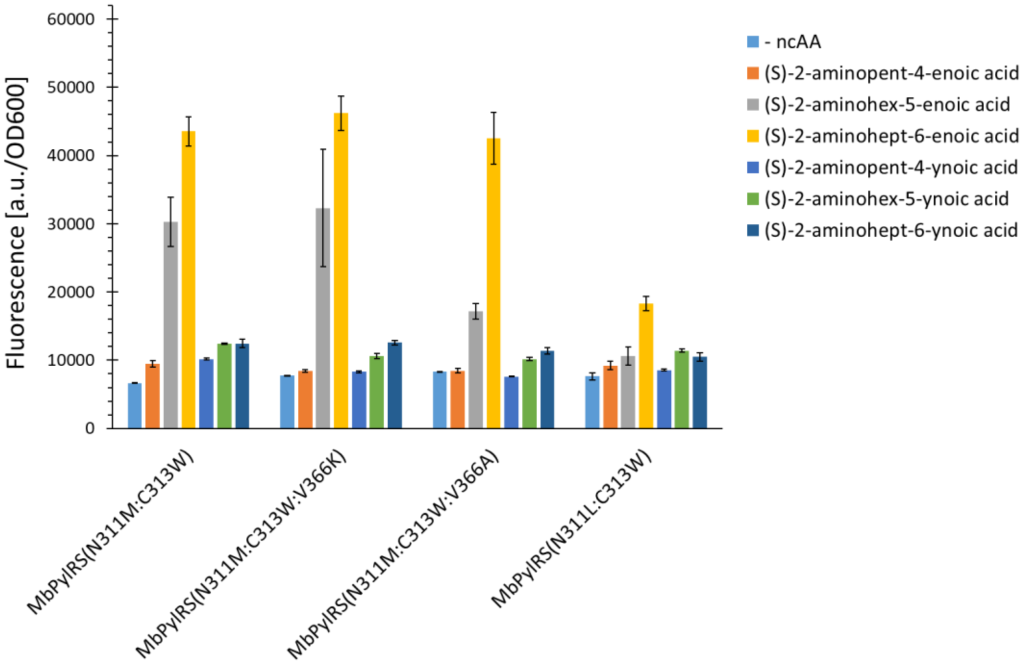

Supplementary Figure S7: Comparison of aliphatic ncAA (**4**, **6**, **8**, **11**, **12**, **13**) incorporation efficiency for *Mb*PylRS variants. Fluorescence measurements of intact *E. coli* BL21(DE3) cells producing the SUMO-sfGFP(R2amber) reporter protein. Intact cell fluorescence of *E. coli* BL21(DE3), with endpoint measurements after 24 h of incubation. The data (incl. standard deviation) represent the mean of three biological replicates.

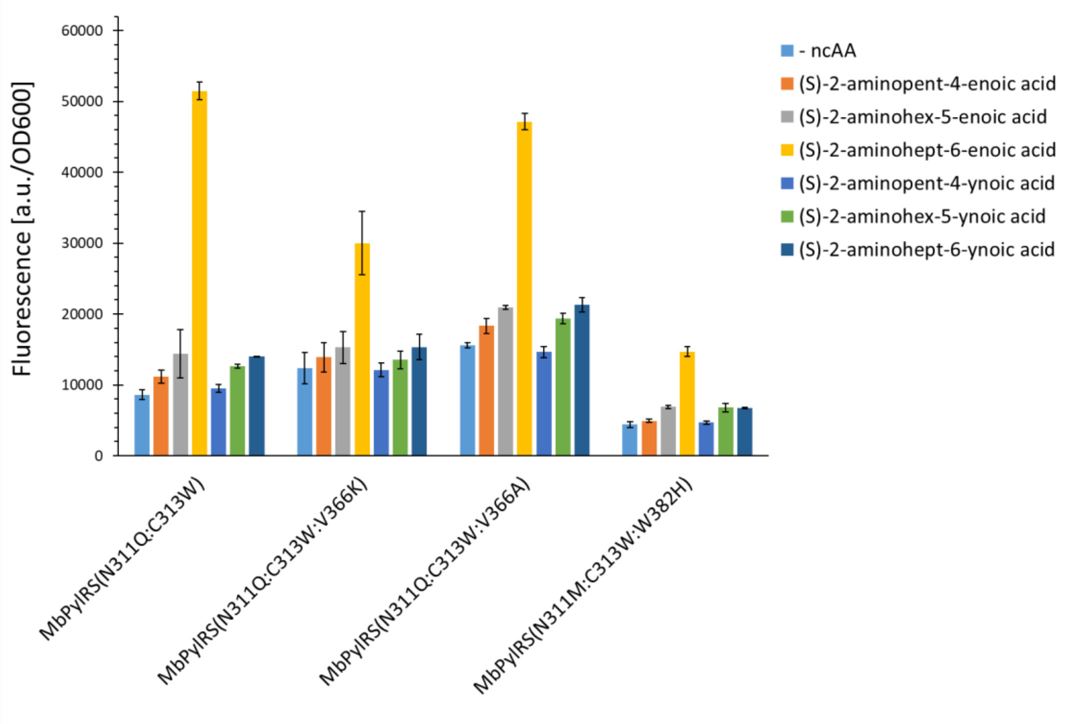

Supplementary Figure S8: Comparison of aliphatic ncAA (**4**, **6**, **8**, **11**, **12**, **13**) incorporation efficiency for *Mb*PylRS variants. Fluorescence measurements of intact *E. coli* BL21(DE3) cells producing the SUMO-sfGFP(R2amber) reporter protein. Intact cell fluorescence of *E. coli* BL21(DE3), with endpoint measurements after 24 h of incubation. The data (incl. standard deviation) represent the mean of three biological replicates.

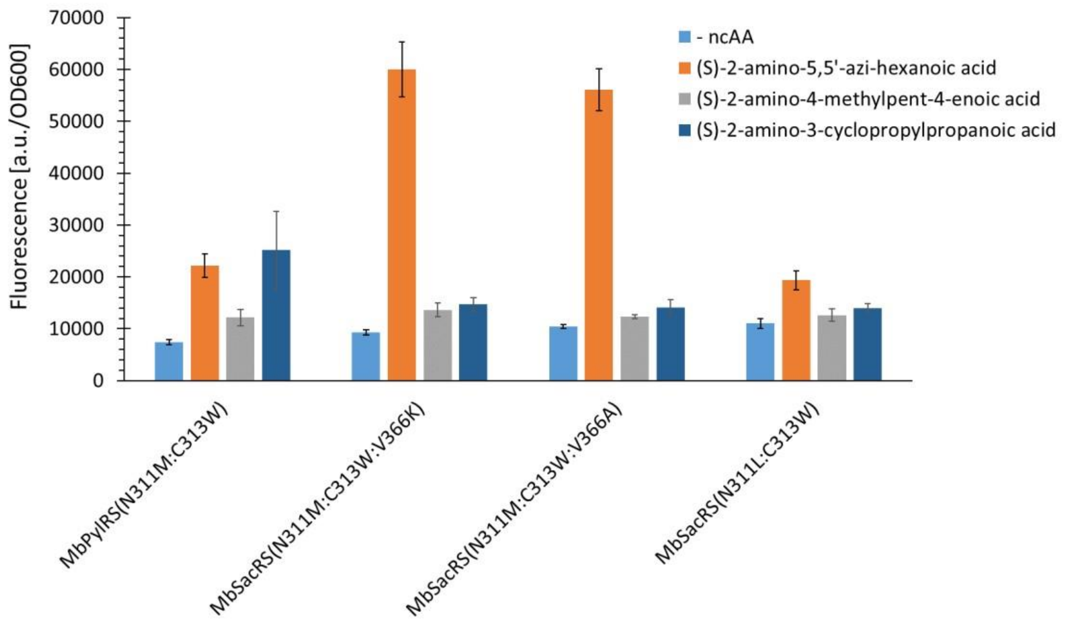

Supplementary Figure S9: Comparison of (*S*)-2-amino-5,5'-azi-hexanoic acid (**20**), (S)-2-amino-4methylpent-4-enoic acid (**21**) and (*S*)-2-amino-3-cyclopropylpropanoic acid (**9**) incorporation efficiency for *Mb*PylRS variants. Fluorescence measurements of intact *E. coli* BL21(DE3) cells producing the SUMO-sfGFP(R2amber) reporter protein. Intact cell fluorescence of *E. coli* BL21(DE3), with endpoint measurements after 24 h of incubation. The data (incl. standard deviation) represent the mean of three biological replicates.

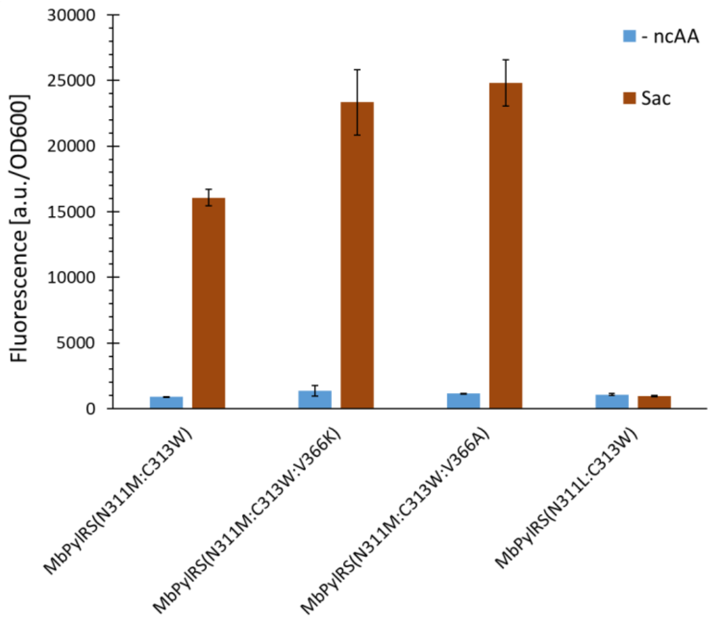

Supplementary Figure S10: Comparison of *S*-allyl-L-cysteine (Sac, **1**) incorporation efficiency for *Mb*PylRS(N311M:C313W) variants mutate at position V366. Fluorescence measurements of intact *E. coli* BL21(DE3) cells producing the SUMO-sfGFP(R2amber) reporter protein. Relative fluorescence is normalized to the highest value. Intact cell fluorescence of *E. coli* BL21(DE3), with endpoint measurements after 24 h of incubation. The data (incl. standard deviation) represent the mean of three biological replicates.

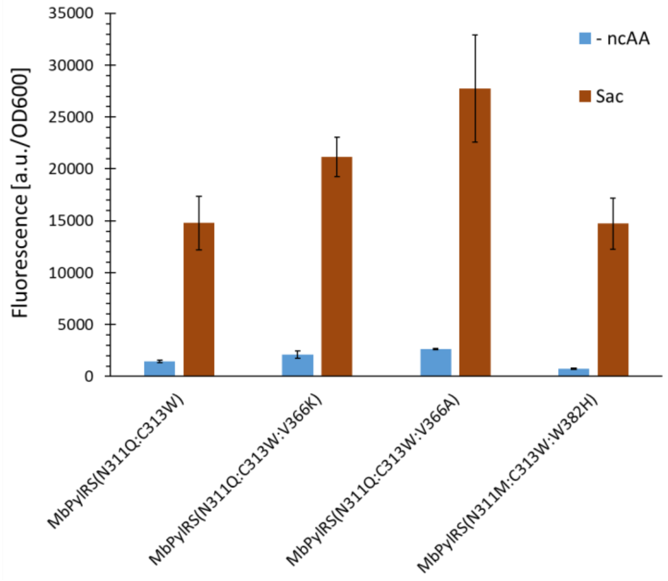

Supplementary Figure S11: Comparison of *S*-allyl-L-cysteine (Sac, **1**) incorporation efficiency for *Mb*PylRS(N311Q:C313W) variants mutate at position V366. Fluorescence measurements of intact *E. coli* BL21(DE3) cells producing the SUMO-sfGFP(R2amber) reporter protein. Relative fluorescence is normalized to the highest value. Intact cell fluorescence of *E. coli* BL21(DE3), with endpoint measurements after 24 h of incubation. The data (incl. standard deviation) represent the mean of three biological replicates.

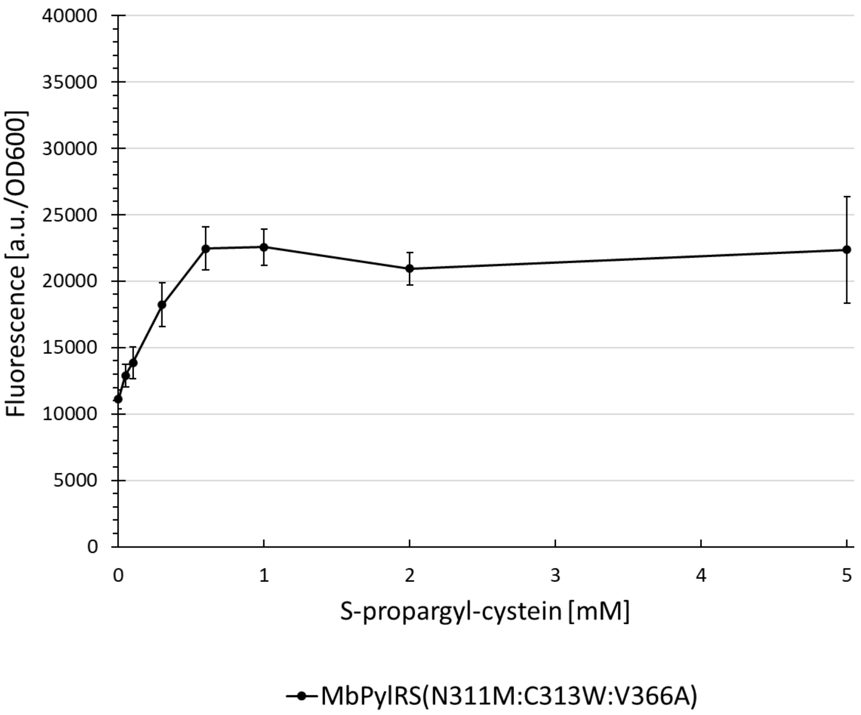

Supplementary Figure S12: Concentration dependent protein production for *Mb*PylRS(N311M:C313W:V366A). Measured fluorescence intensity of intact *E. coli* BL21(DE3) cells expressing the sfGFP(R2 amber) reporter, with endpoint measurements after 24 h. Endpoint measurements comprise different ncAA concentrations (0.05, 0.1, 0.3, 0.6, 1, 2, and 5 mM) of *S*propargyl-L-cysteine (**27**). The data (incl. standard deviation) represent the mean of three biological replicates.

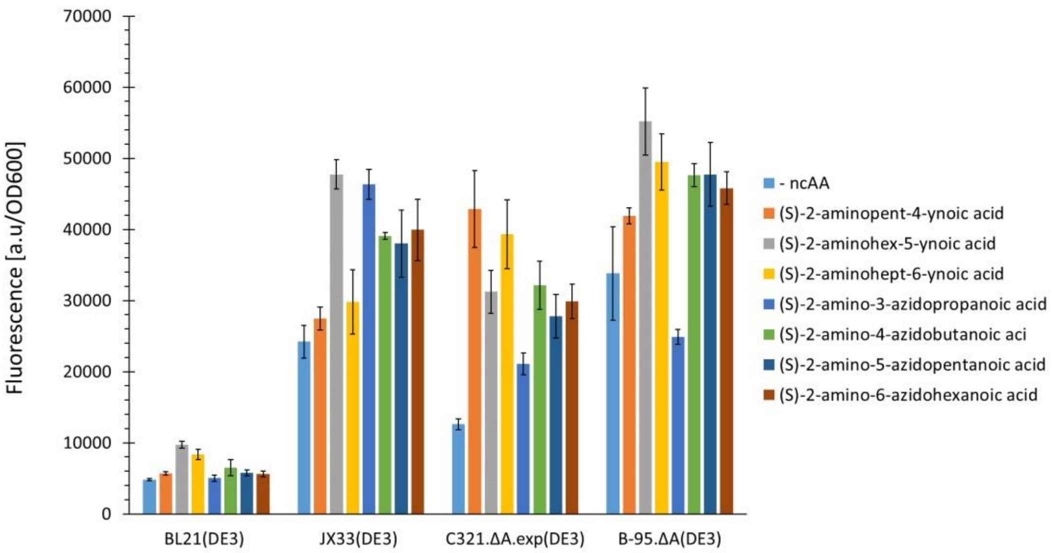

Supplementary Figure S13: Comparison of ncAA (**11**, **12**, **13**, **14**, **15**, **16**, **17**) incorporation efficiency for *Mb*PylRS(N311M:C313W) in different *E. coli* host strains. Fluorescence measurements of intact *E. coli* BL21(DE3) cells producing the SUMO-sfGFP(R2amber) reporter protein. Intact cell fluorescence of *E. coli* BL21(DE3), endpoint measurements after 24 h of incubation. The data (incl. standard deviation) represent the mean of three biological replicates.

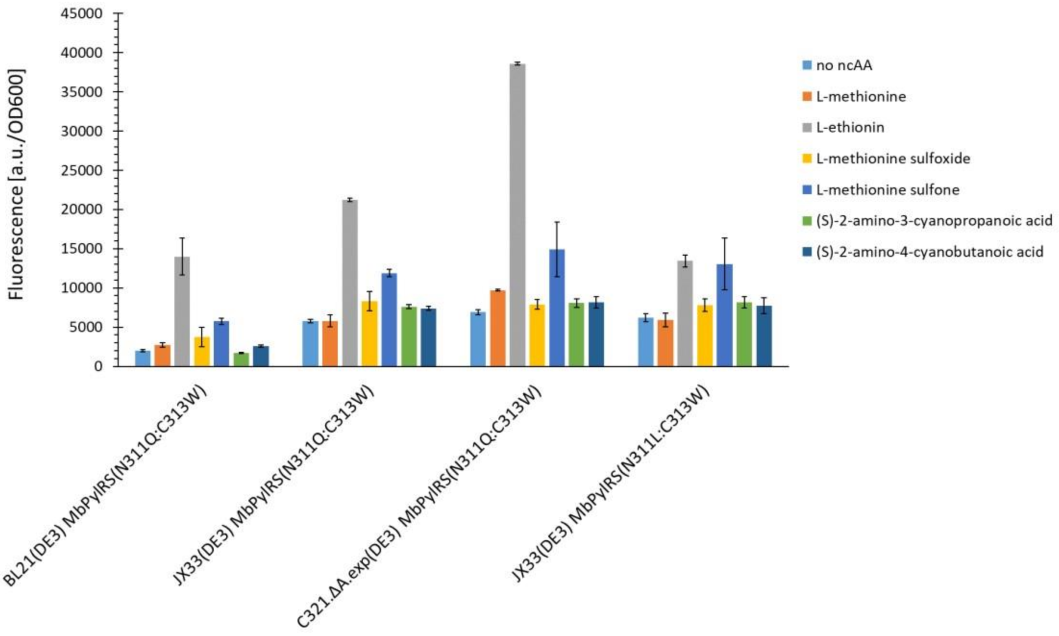

Supplementary Figure S14: Comparison of ncAA (**18**, **19**, **22**, **23**, **24**, **25**) incorporation efficiency for *Mb*PylRS constructs in different *E. coli* host strains. Fluorescence measurements of intact *E. coli* BL21(DE3) cells producing the SUMO-sfGFP(R2amber) reporter protein. Intact cell fluorescence of *E. coli* BL21(DE3), endpoint measurements after 24 h of incubation. The data (incl. standard deviation) represent the mean of three biological replicates.

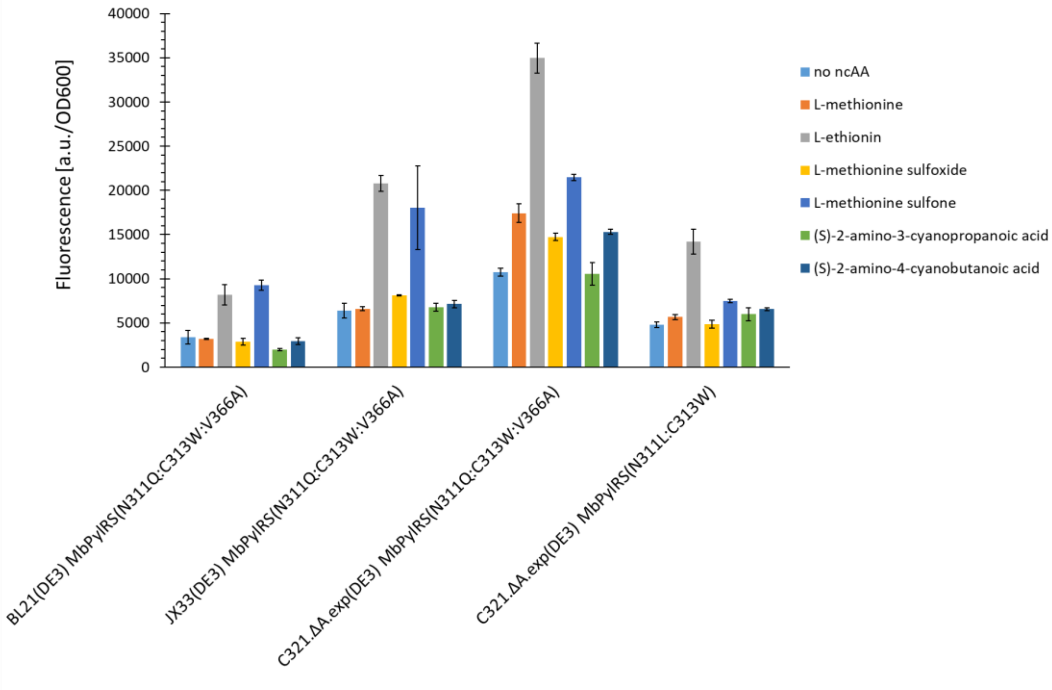

Supplementary Figure S15: Comparison of ncAA (**18**, **19**, **22**, **23**, **24**, **25**) incorporation efficiency for *Mb*PylRS constructs in different *E. coli* host strains. Fluorescence measurements of intact *E. coli* BL21(DE3) cells producing the SUMO-sfGFP(R2amber) reporter protein. Intact cell fluorescence of *E. coli* BL21(DE3), with endpoint measurements after 24 h of incubation. The data (incl. standard deviation) represent the mean of three biological replicates.

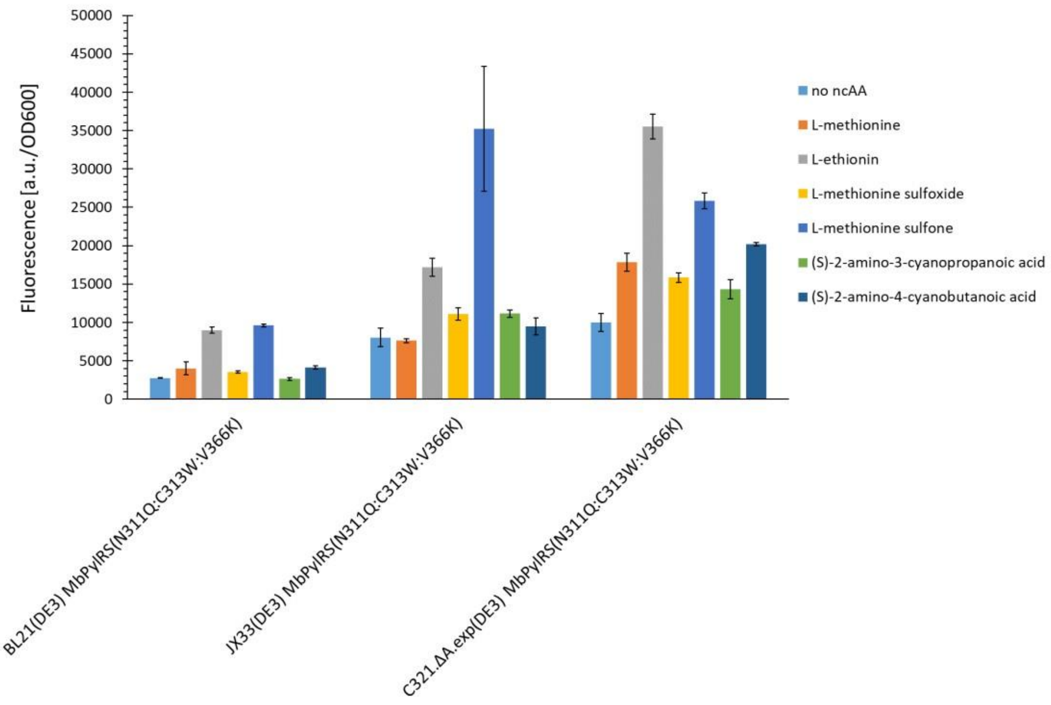

Supplementary Figure S16: Comparison of ncAA (**18**, **19**, **22**, **23**, **24**, **25**) incorporation efficiency for *Mb*PylRS constructs in different *E. coli* host strains. Fluorescence measurements of intact *E. coli* BL21(DE3) cells producing the SUMO-sfGFP(R2amber) reporter protein. Intact cell fluorescence of *E. coli* BL21(DE3), with endpoint measurements after 24 h of incubation. The data (incl. standard deviation) represent the mean of three biological replicates.

2.3. Mass Spectrometry Data

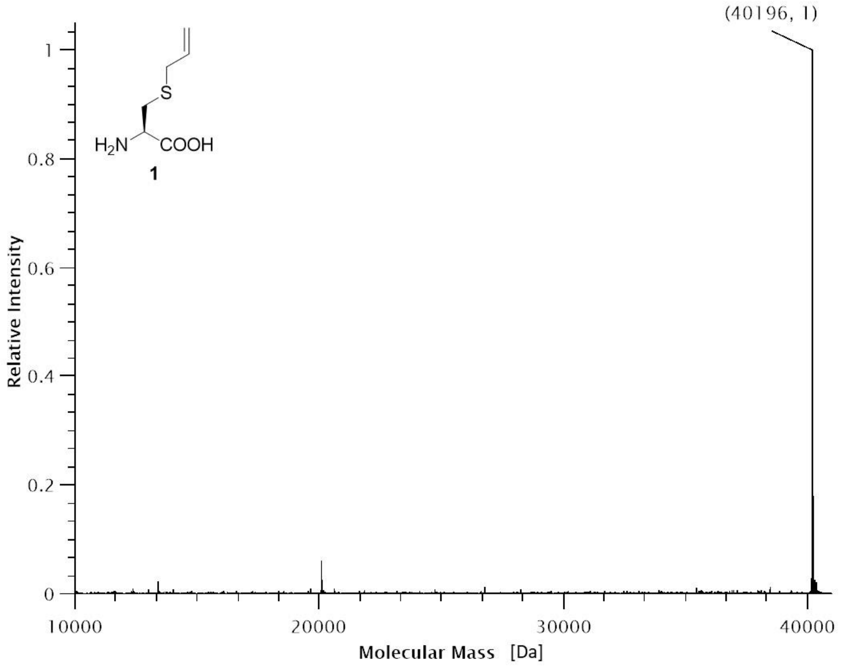

Supplementary Figure S17: Deconvoluted ESI-MS spectrum of His6-SUMO-sfGFP(R2(**ncAA**))-Strep production in *E. coli* BL21(DE3) with co-expression of SmbP-*Mb*SacRS(N311M:C313W:V366A). Expected protein mass: 40194.9 Da. Observed mass: 40196 Da.

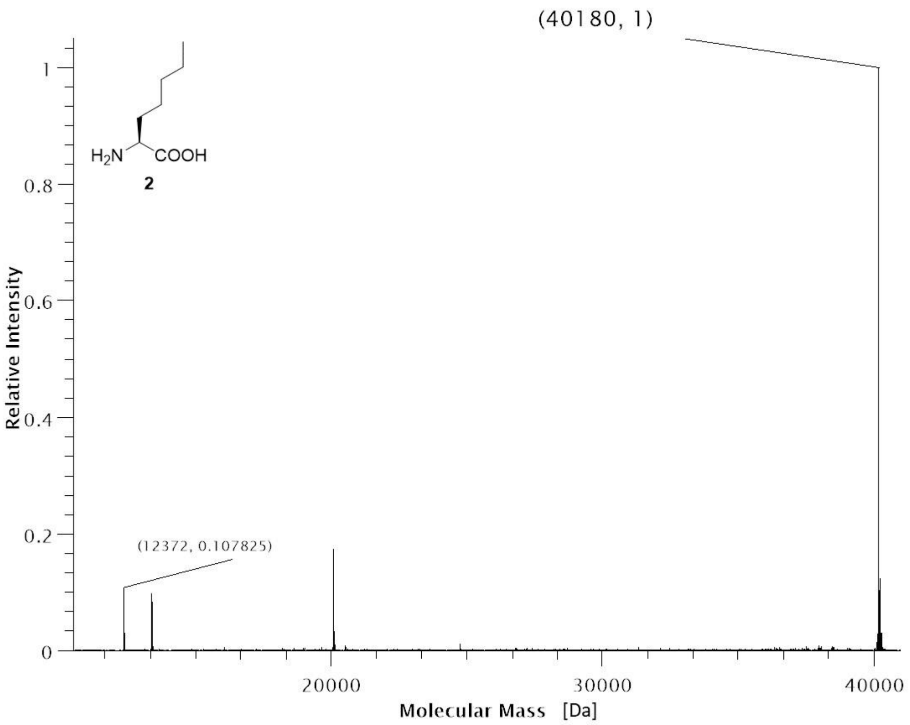

Supplementary Figure S18: Deconvoluted ESI-MS spectrum of His6-SUMO-sfGFP(R2(**ncAA**))-Strep production in *E. coli* BL21(DE3) with co-expression of SmbP-*Mb*SacRS(N311Q:C313W). Expected protein mass: 40178.8 Da. Observed mass: 40180 Da. Expected mass of His6-SUMO truncation product: 12372.8 Da. Observed mass: 12372 Da.

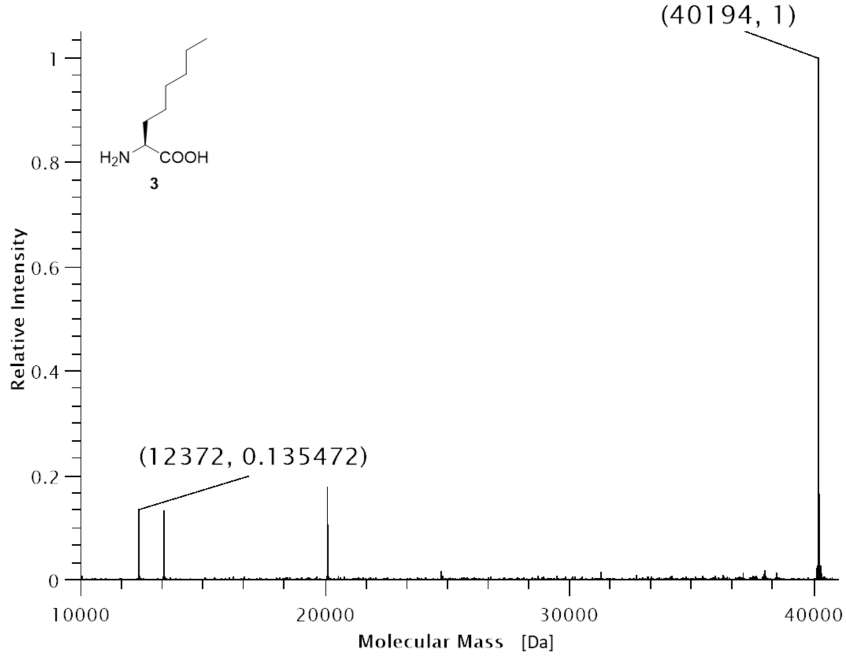

Supplementary Figure S19: Deconvoluted ESI-MS spectrum of His6-SUMO-sfGFP(R2(**ncAA**))-Strep production in *E. coli* BL21(DE3) with co-expression of SmbP-*Mb*SacRS(N311M:C313W:V366A). Expected protein mass: 40192.9 Da. Observed mass: 40194 Da. Expected mass of His6-SUMO truncation product: 12372.8 Da. Observed mass: 12372 Da.

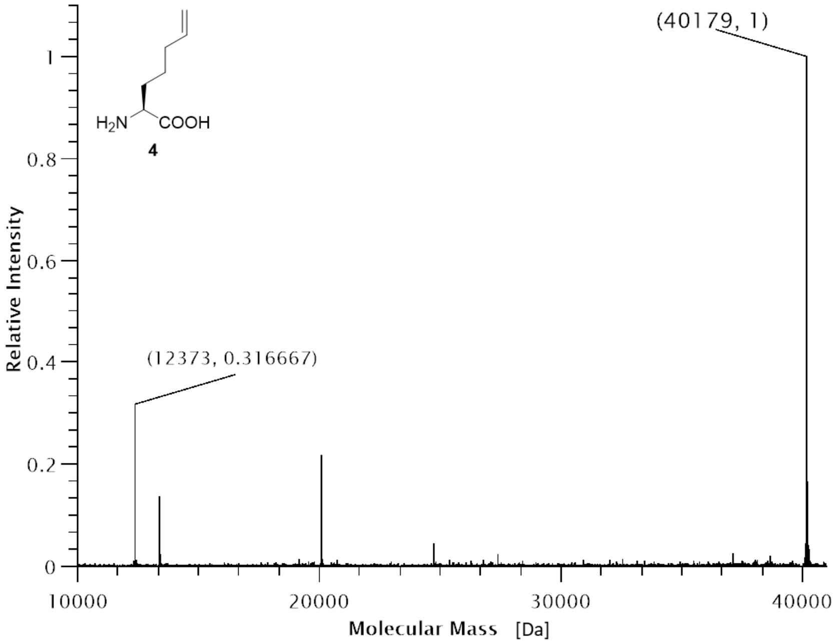

Supplementary Figure S20: Deconvoluted ESI-MS spectrum of His$_6$-SUMO-sfGFP(R2(**ncAA**))-Strep production in *E. coli* BL21(DE3) with co-expression of SmbP-*Mb*SacRS(N311M:C313W:V366A). Expected protein mass: 40176.8 Da. Observed mass: 40179 Da. Expected mass of His$_6$-SUMO truncation product: 12372.8 Da. Observed mass: 12373 Da.

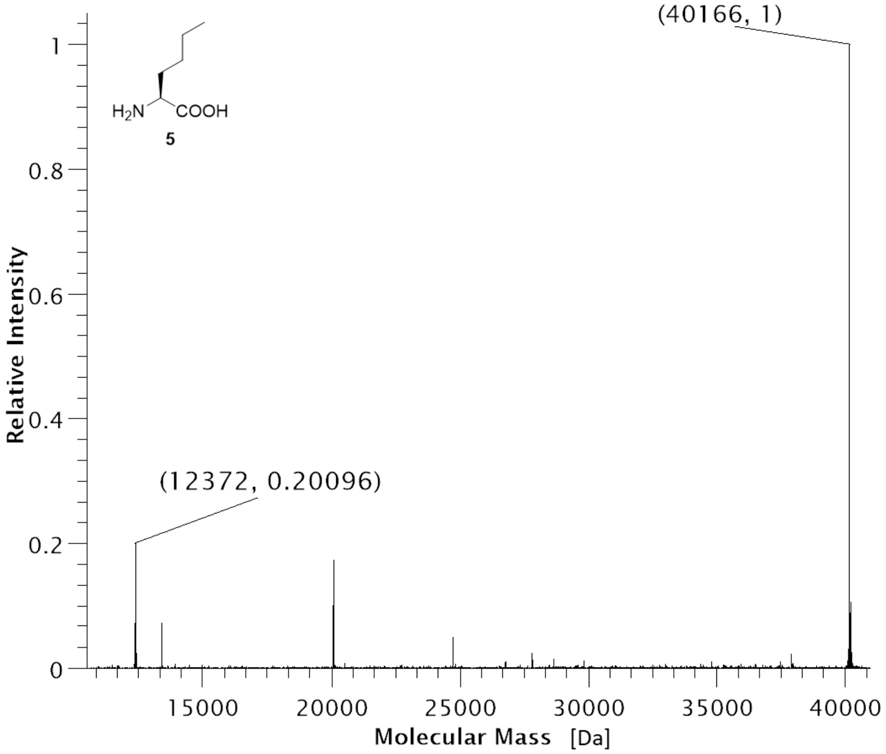

Supplementary Figure S21: Deconvoluted ESI-MS spectrum of His$_6$-SUMO-sfGFP(R2(**ncAA**))-Strep production in *E. coli* BL21(DE3) with co-expression of SmbP-*Mb*SacRS(N311M:C313W). Expected protein mass: 40164.8 Da. Observed mass: 40166 Da. Expected mass of His$_6$-SUMO truncation product: 12372.8 Da. Observed mass: 12372 Da.

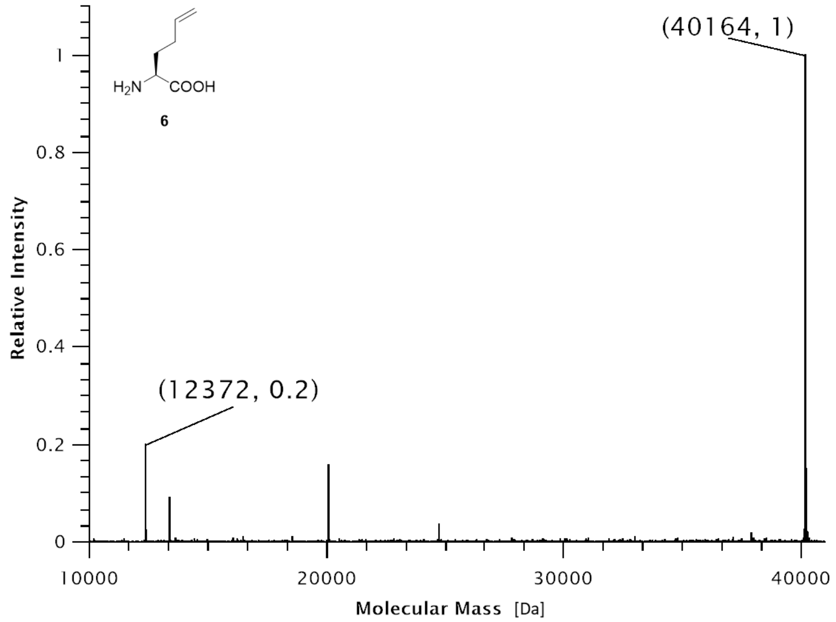

Supplementary Figure S22: Deconvoluted ESI-MS spectrum of His6-SUMO-sfGFP(R2(**ncAA**))-Strep production in *E. coli* BL21(DE3) with co-expression of SmbP-*Mb*SacRS(N311M:C313W:V366K). Expected protein mass: 40162.8 Da. Observed mass: 40164 Da. Expected mass of His6-SUMO truncation product: 12372.8 Da. Observed mass: 12372 Da.

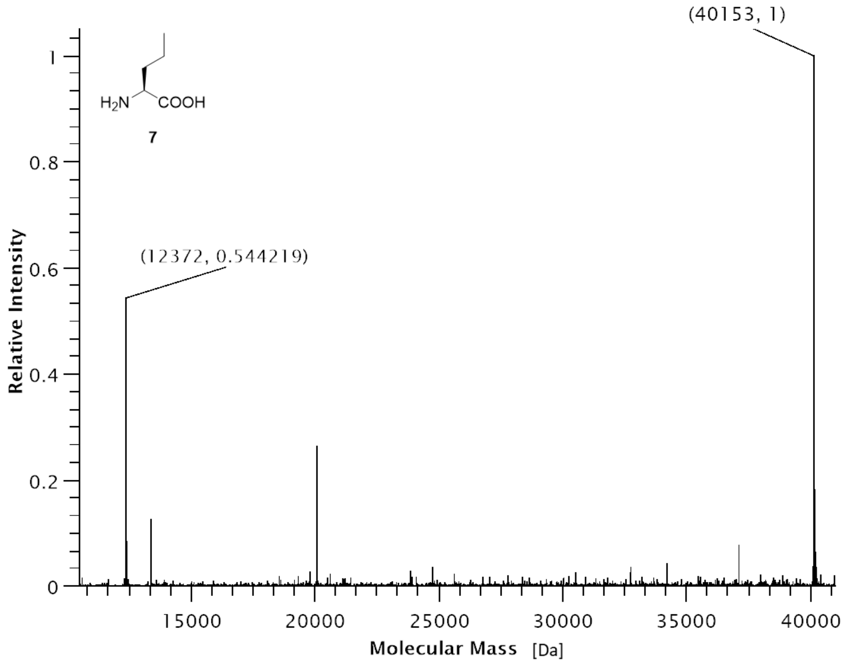

Supplementary Figure S23: Deconvoluted ESI-MS spectrum of His6-SUMO-sfGFP(R2(**ncAA**))-Strep production in *E. coli* BL21(DE3) with co-expression of SmbP-*Mb*SacRS(N311M:C313W). Expected protein mass: 40150.8 Da. Observed mass: 40153 Da. Expected mass of His6-SUMO truncation product: 12372.8 Da. Observed mass: 12372 Da.

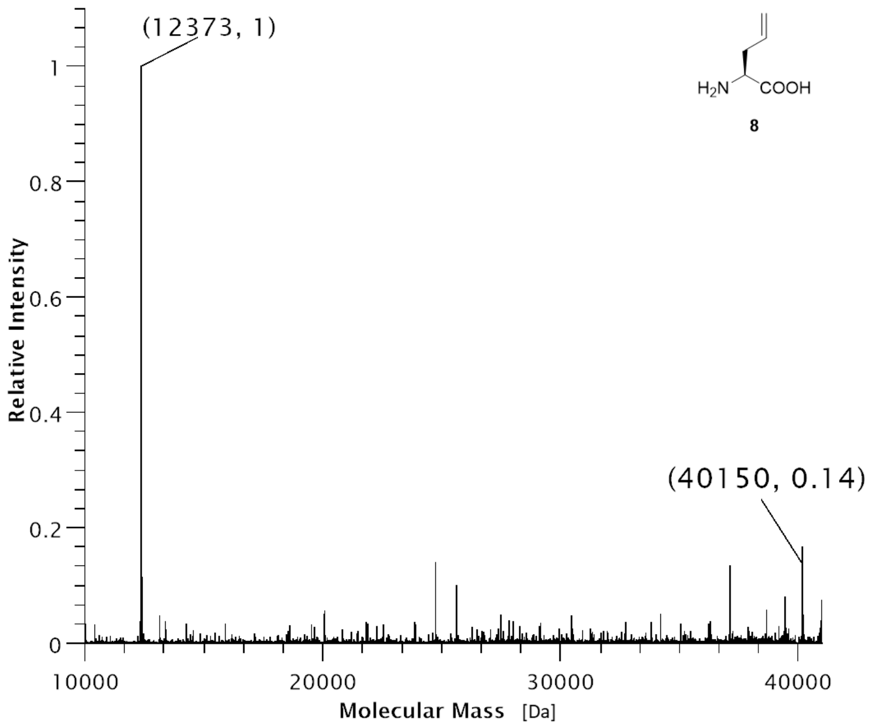

Supplementary Figure S24: Deconvoluted ESI-MS spectrum of His6-SUMO-sfGFP(R2(**ncAA**))-Strep production in *E. coli* BL21(DE3) with co-expression of SmbP-*Mb*SacRS(N311M:C313W). Expected protein mass: 40148.8 Da. Observed mass: 40150 Da. Expected mass of His6-SUMO truncation product: 12372.8 Da. Observed mass: 12373 Da.

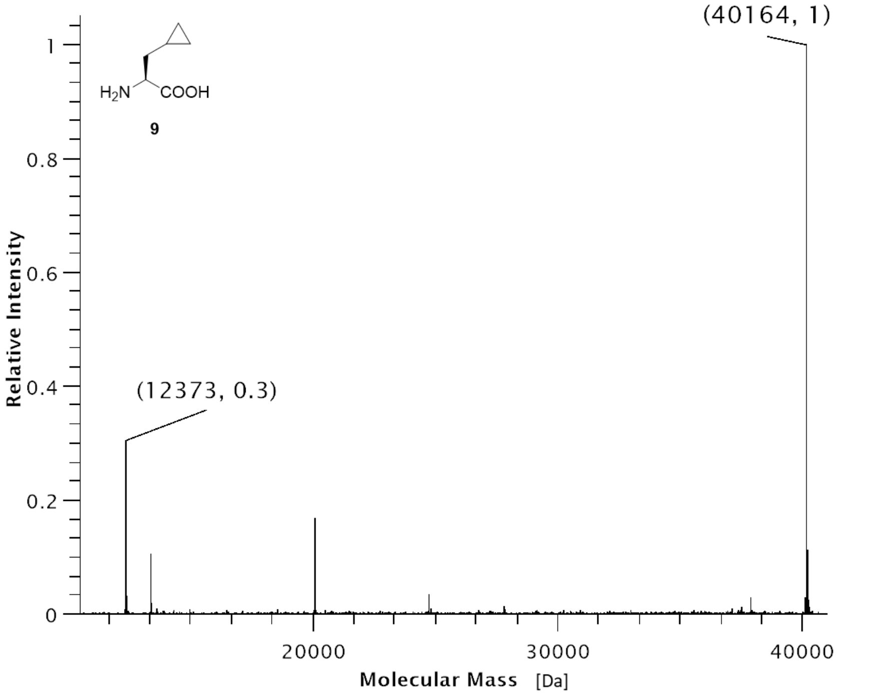

Supplementary Figure S25: Deconvoluted ESI-MS spectrum of His6-SUMO-sfGFP(R2(**ncAA**))-Strep production in *E. coli* BL21(DE3) with co-expression of SmbP-*Mb*SacRS(N311M:C313W). Expected protein mass: 40162.8 Da. Observed mass: 40164 Da. Expected mass of His6-SUMO truncation product: 12372.8 Da. Observed mass: 12373 Da.

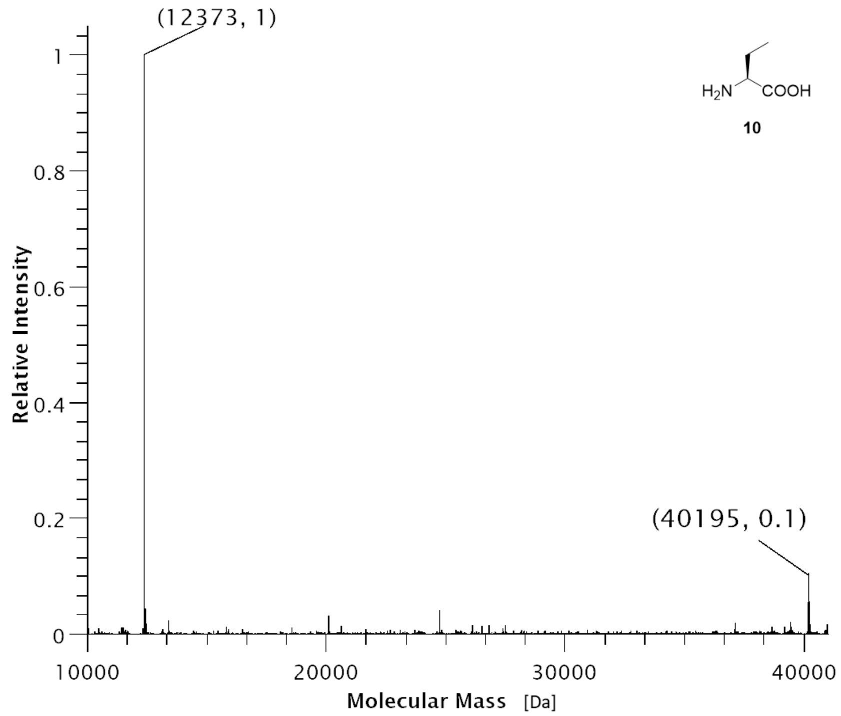

Supplementary Figure S26: Deconvoluted ESI-MS spectrum of His6-SUMO-sfGFP(R2(**ncAA**))-Strep production in *E. coli* BL21(DE3) with co-expression of SmbP-*Mb*SacRS(N311M:C313W:V366A). Expected protein mass: 40136.8 Da. Observed mass: 40195 Da. Expected mass of His6-SUMO truncation product: 12372.8 Da. Observed mass: 12373 Da.

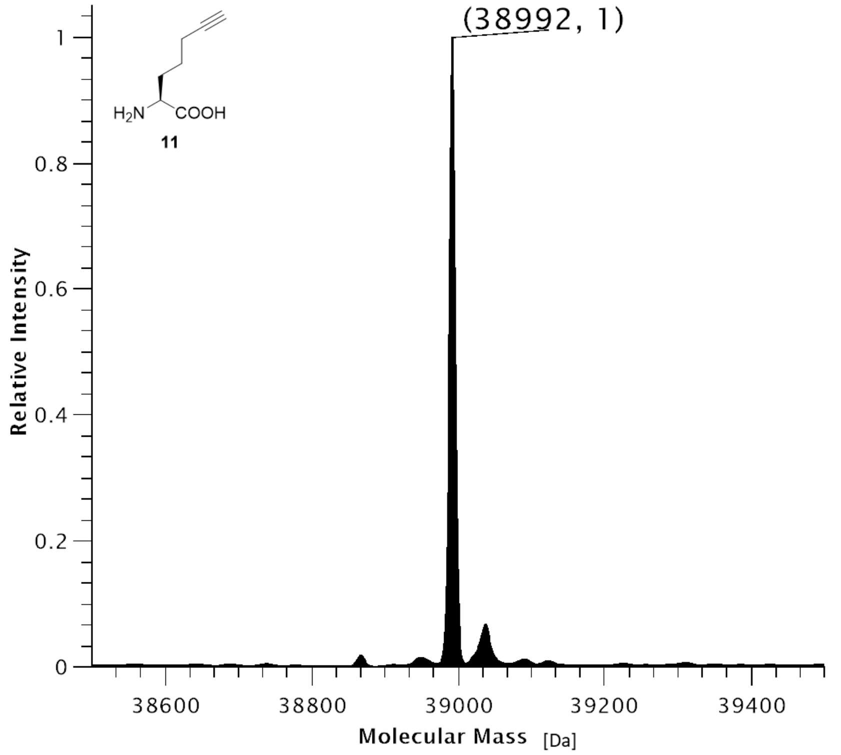

Supplementary Figure S27: Deconvoluted ESI-MS spectrum of SUMO-sfGFP(R2(**ncAA**))-His6 production in *E. coli* C321.ΔA.exp(DE3) with co-expression of SmbP-*Mb*SacRS(N311M:C313W). Expected protein mass: 38990.9 Da. Observed mass: 38992 Da.

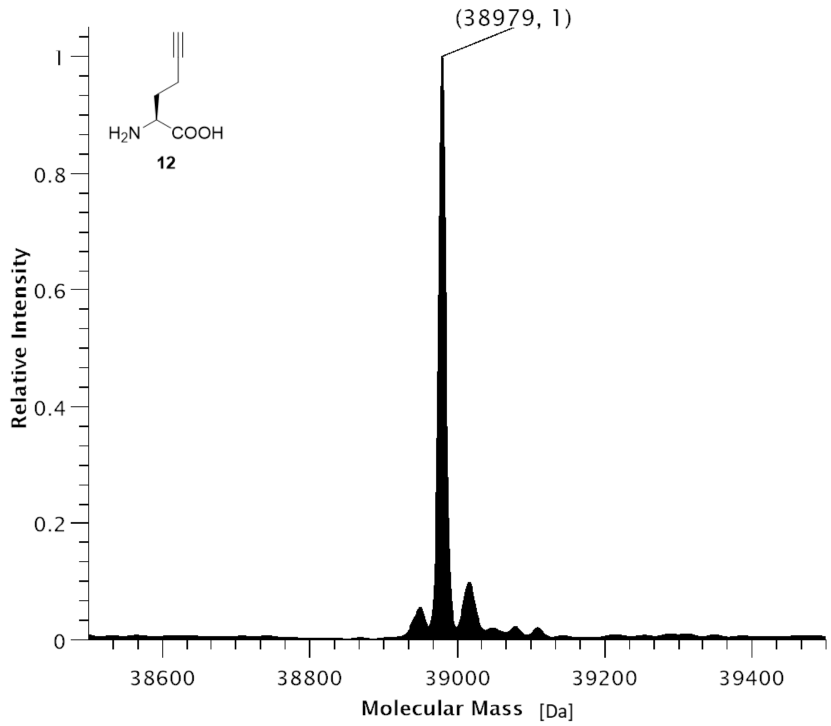

Supplementary Figure S28: Deconvoluted ESI-MS spectrum of SUMO-sfGFP(R2(**ncAA**))-His6 production in *E. coli* C321.ΔA.exp(DE3) with co-expression of SmbP-*Mb*SacRS(N311M:C313W). Expected protein mass: 38976.9 Da. Observed mass: 38979 Da.

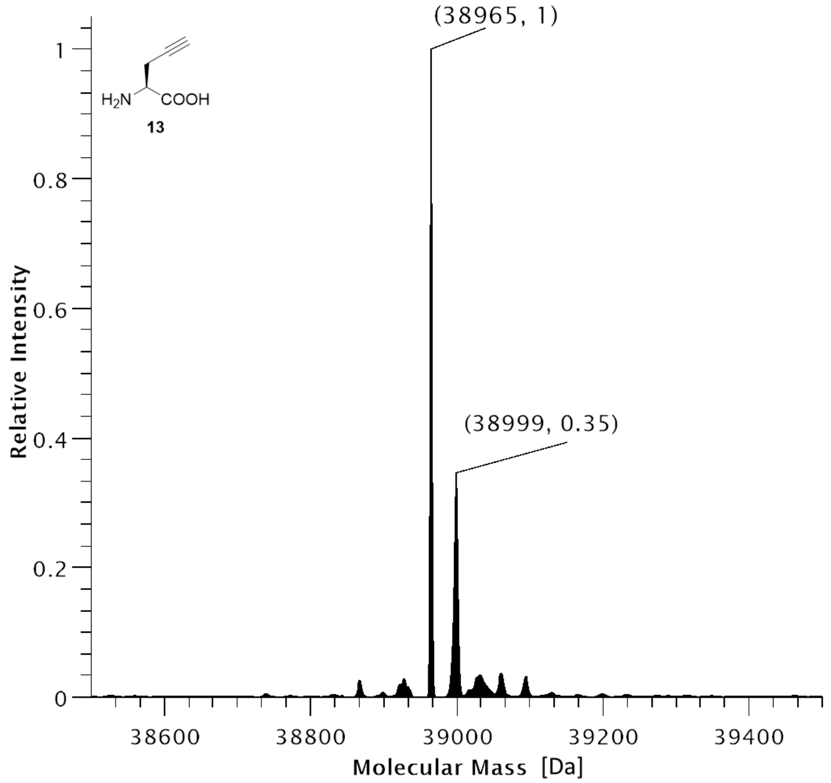

Supplementary Figure S29: Deconvoluted ESI-MS spectrum of SUMO-sfGFP(R2(**ncAA**))-His6 production in *E. coli* C321.ΔA.exp(DE3) with co-expression of SmbP-*Mb*SacRS(N311M:C313W). Expected protein mass: 38962.8 Da. Observed mass: 38965 Da.

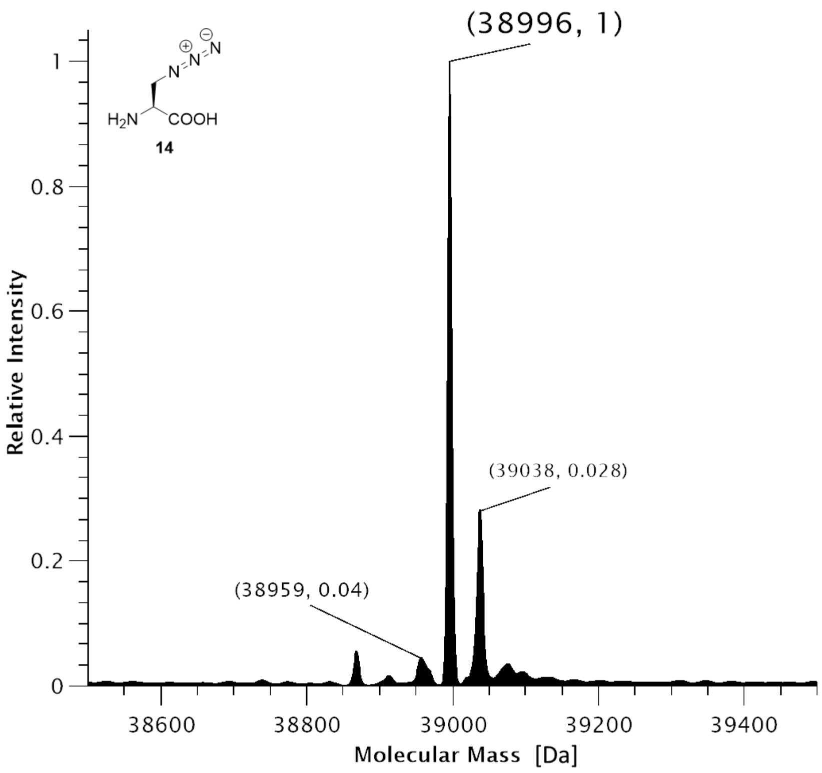

Supplementary Figure S30: Deconvoluted ESI-MS spectrum of SUMO-sfGFP(R2(**ncAA**))-His_6 production in *E. coli* JX33(DE3) with co-expression of SmbP-*Mb*SacRS(N311M:C313W). Expected protein mass: 38979.8 Da. Observed mass: 38996 Da.

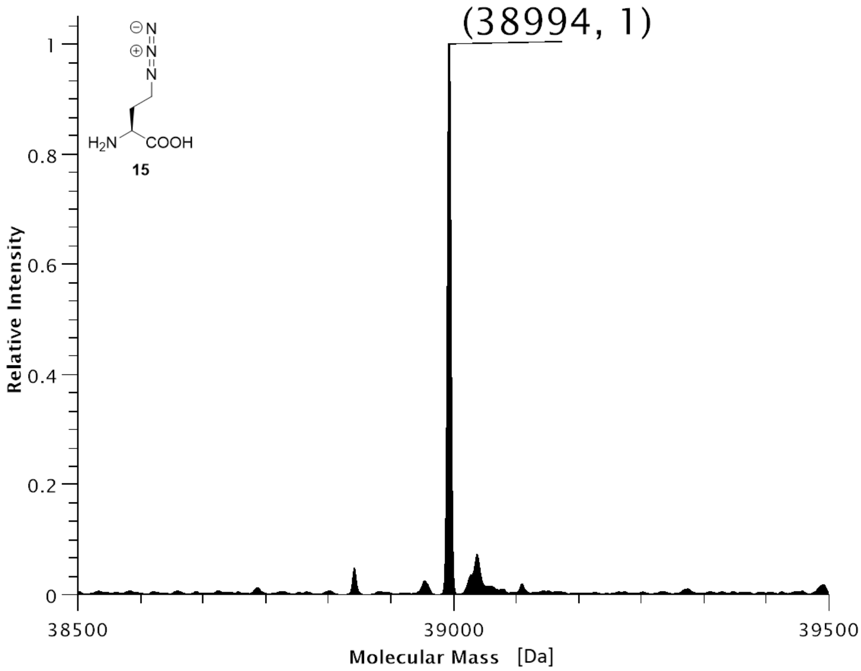

Supplementary Figure S31: Deconvoluted ESI-MS spectrum of SUMO-sfGFP(R2(**ncAA**))-His_6 production in *E. coli* C321.ΔA.exp(DE3) with co-expression of SmbP-*Mb*SacRS(N311M:C313W). Expected protein mass: 38993.8 Da. Observed mass: 38994 Da.

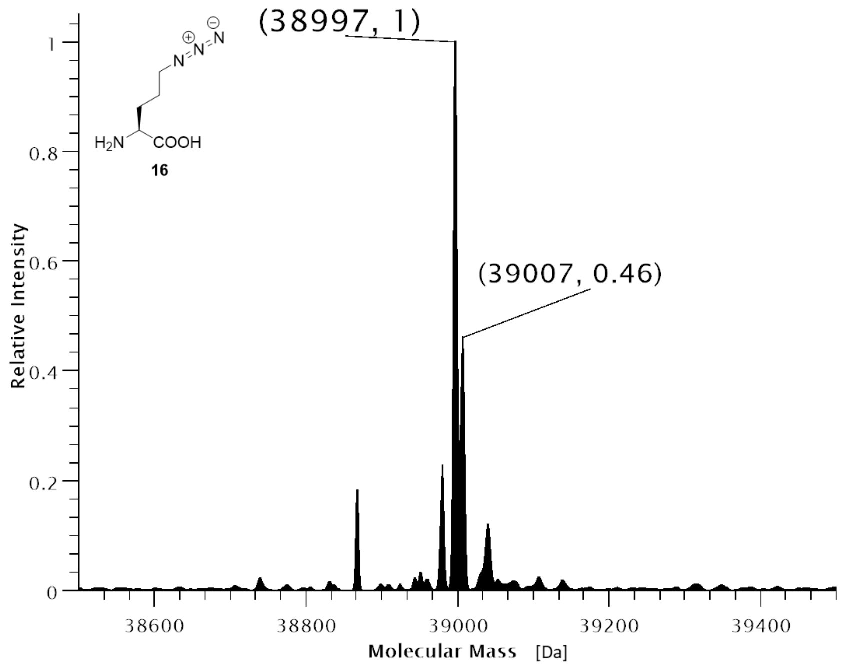

Supplementary Figure S32: Deconvoluted ESI-MS spectrum of SUMO-sfGFP(R2(**ncAA**))-His6 production in *E. coli* C321.ΔA.exp(DE3) with co-expression of SmbP-*Mb*SacRS(N311M:C313W). Expected protein mass: 39007.9 Da. Observed mass: 39007 Da.

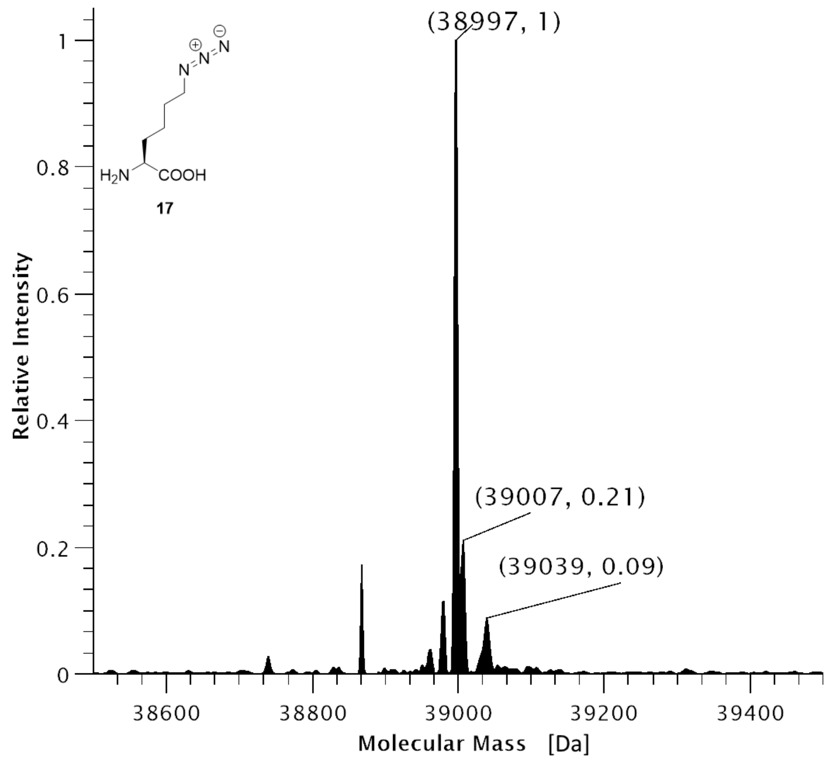

Supplementary Figure S33: Deconvoluted ESI-MS spectrum of SUMO-sfGFP(R2(**ncAA**))-His6 production in *E. coli* C321.ΔA.exp(DE3) with co-expression of SmbP-*Mb*SacRS(N311M:C313W). Expected protein mass: 39021.9 Da. Observed mass: 38997 Da.

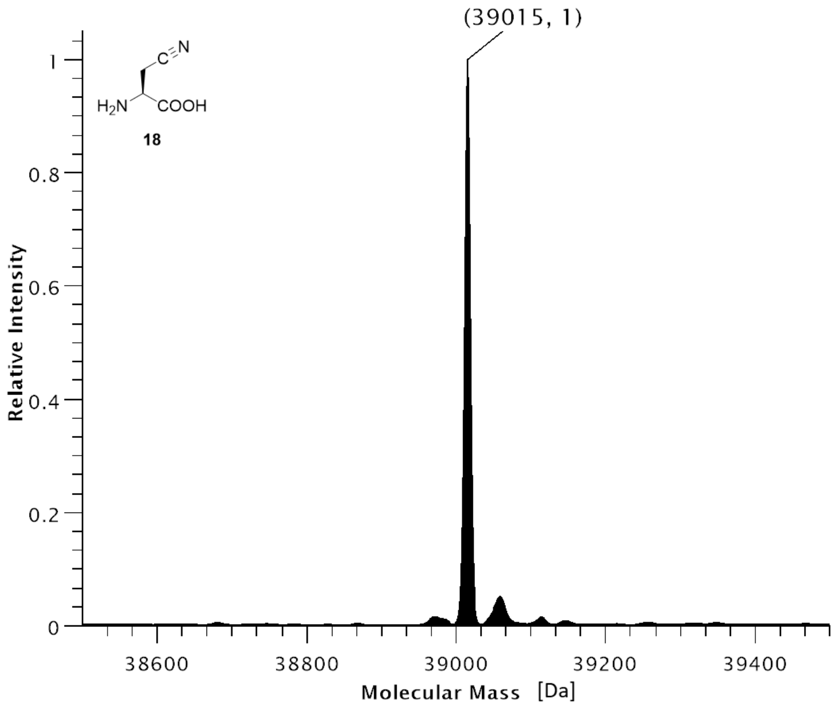

Supplementary Figure S34: Deconvoluted ESI-MS spectrum of SUMO-sfGFP(R2(**ncAA**))-His_6 production in *E. coli* C321.ΔA.exp(DE3) with co-expression of SmbP-*Mb*SacRS(N311Q:C313W:V366K). Expected protein mass: 38963.8 Da. Observed mass: 39015 Da.

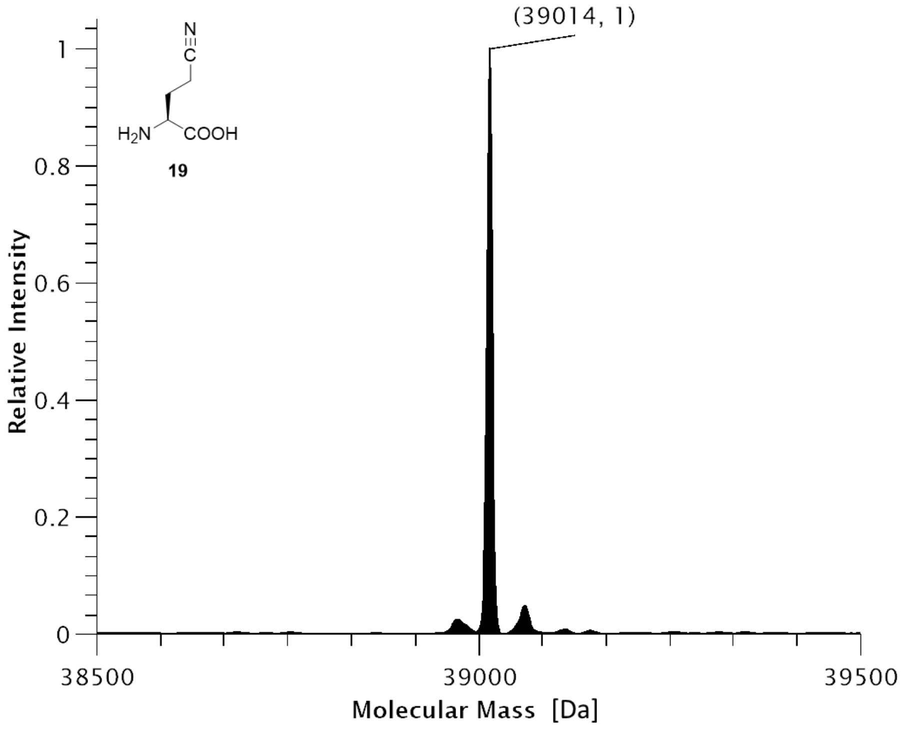

Supplementary Figure S35: Deconvoluted ESI-MS spectrum of SUMO-sfGFP(R2(**ncAA**))-His_6 production in *E. coli* C321.ΔA.exp(DE3) with co-expression of SmbP-*Mb*SacRS(N311Q:C313W:V366K). Expected protein mass: 38977.8 Da. Observed mass: 39014 Da.

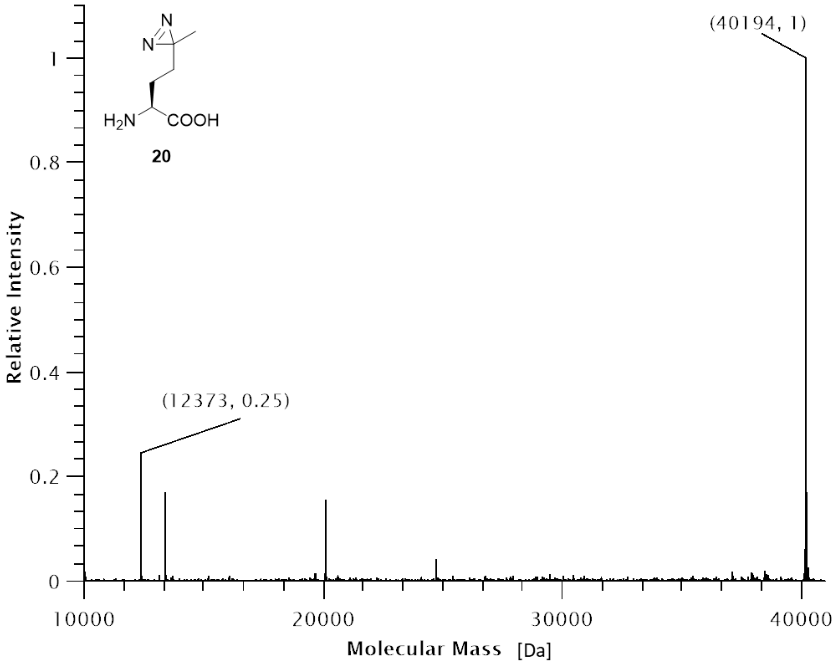

Supplementary Figure S36: Deconvoluted ESI-MS spectrum of His6-SUMO-sfGFP(R2(**ncAA**))-Strep production in *E. coli* BL21(DE3) with co-expression of SmbP-*Mb*SacRS(N311M:C313W:V366A). Expected protein mass: 40190.8 Da. Observed mass: 40194 Da. Expected mass of His6-SUMO truncation product: 12372.8 Da. Observed mass: 12373 Da.

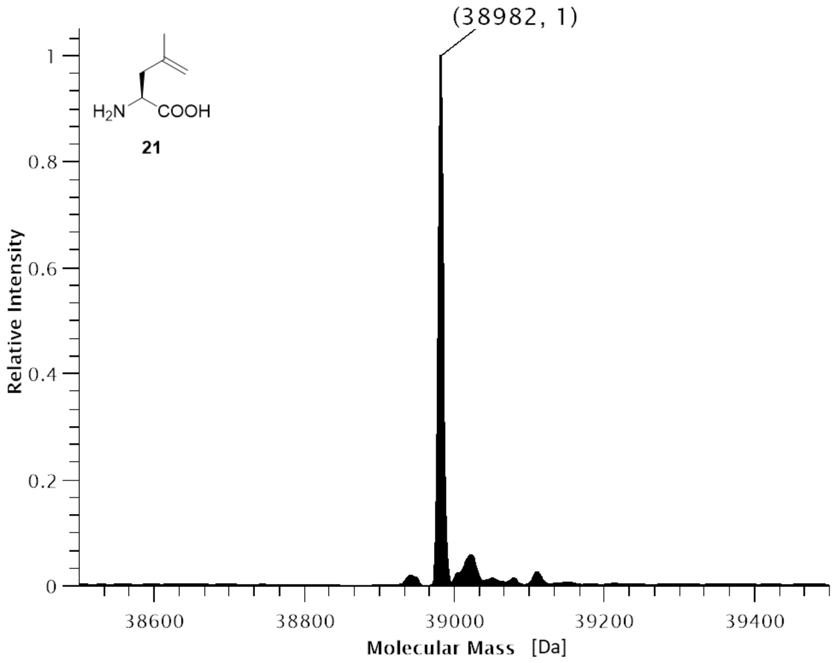

Supplementary Figure S37: Deconvoluted ESI-MS spectrum of SUMO-sfGFP(R2(**ncAA**))-His6 production in *E. coli* BL21(DE3) with co-expression of SmbP-*Mb*SacRS(N311M:C313W). Expected protein mass: 38978.9 Da. Observed mass: 38982 Da.

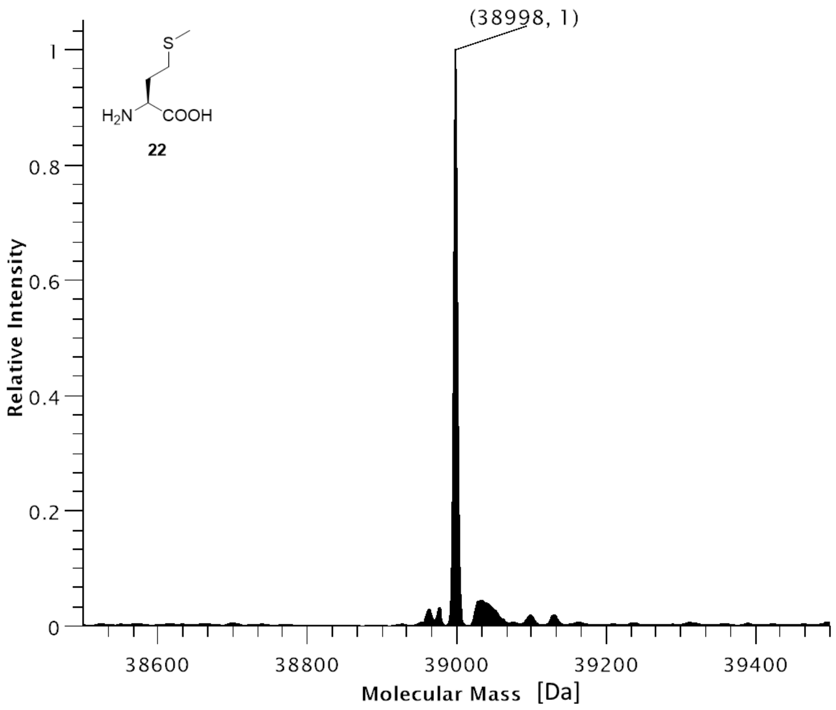

Supplementary Figure S38: Deconvoluted ESI-MS spectrum of SUMO-sfGFP(R2(**AA**))-His6 production in *E. coli* BL21(DE3) with co-expression of SmbP-*Mb*SacRS(N311M:C313W). Expected protein mass: 38998.9 Da. Observed mass: 38998 Da.

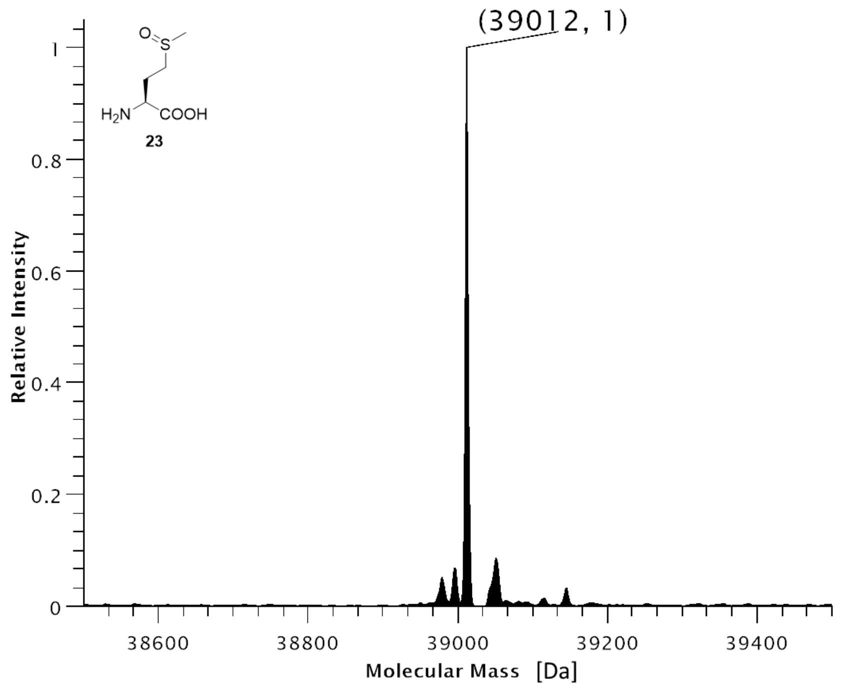

Supplementary Figure S39: Deconvoluted ESI-MS spectrum of SUMO-sfGFP(R2(**ncAA**))-His6 production in *E. coli* BL21(DE3) with co-expression of SmbP-*Mb*SacRS(N311Q:C313W). Expected protein mass: 39014.9 Da. Observed mass: 39012 Da.

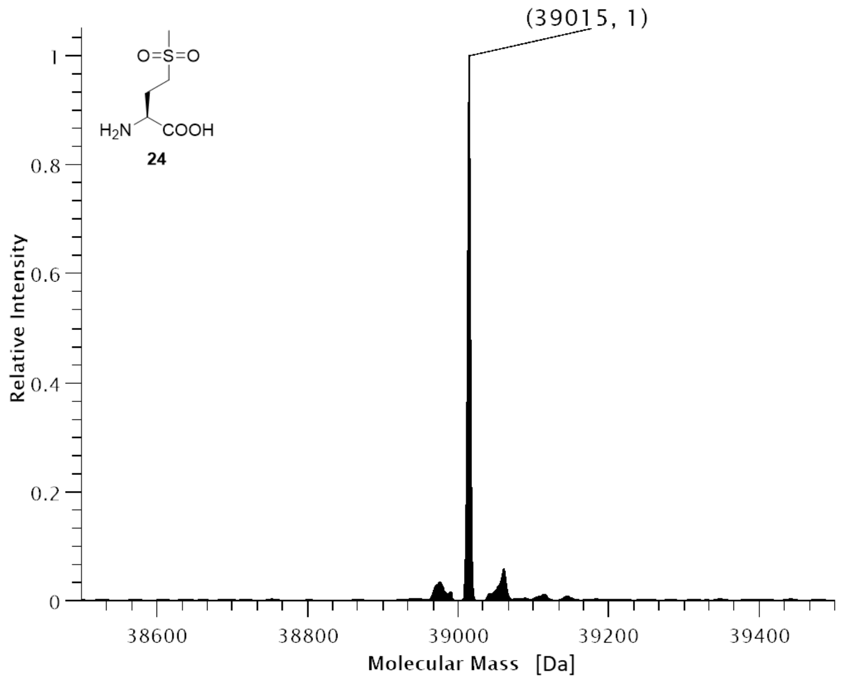

Supplementary Figure S40: Deconvoluted ESI-MS spectrum of SUMO-sfGFP(R2(**ncAA**))-His6 production in *E. coli* BL21(DE3) with co-expression of SmbP-*Mb*SacRS(N311M:C313W:V366K). Expected protein mass: 39030.9 Da. Observed mass: 39015 Da.

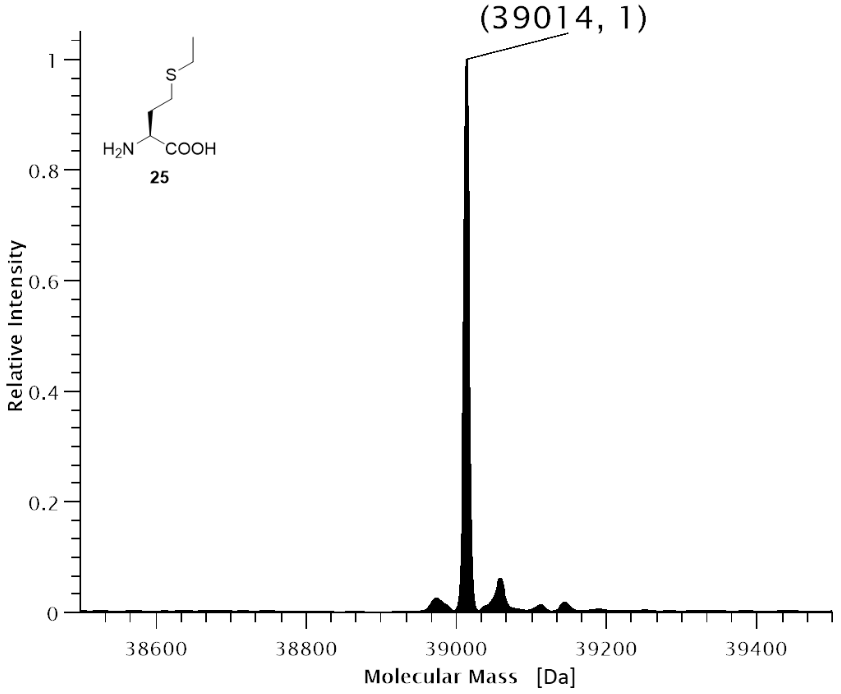

Supplementary Figure S41: Deconvoluted ESI-MS spectrum of SUMO-sfGFP(R2(**ncAA**))-His6 production in *E. coli* BL21(DE3) with co-expression of SmbP-*Mb*SacRS(N311Q:C313W). Expected protein mass: 39013 Da. Observed mass: 39014 Da.

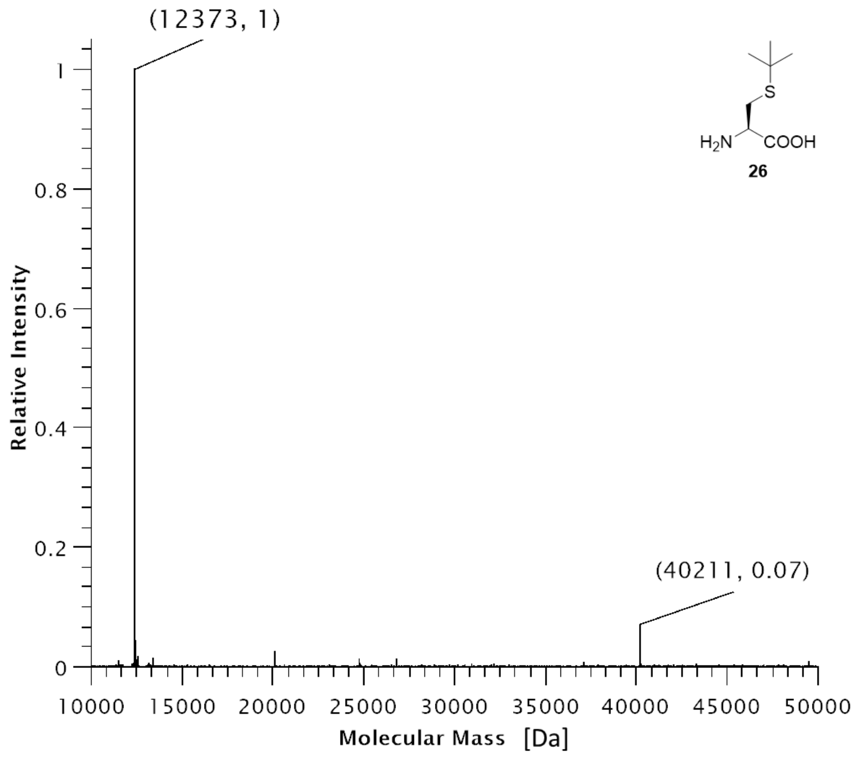

Supplementary Figure S42: Deconvoluted ESI-MS spectrum of His6-SUMO-sfGFP(R2(**ncAA**))-Strep production in *E. coli* BL21(DE3) with co-expression of SmbP-*Mb*SacRS(N311M:C313W:V366A). Expected protein mass: 40190.8 Da. Observed mass: 40194 Da. Expected mass of His6-SUMO truncation product: 12372.8 Da. Observed mass: 12373 Da.

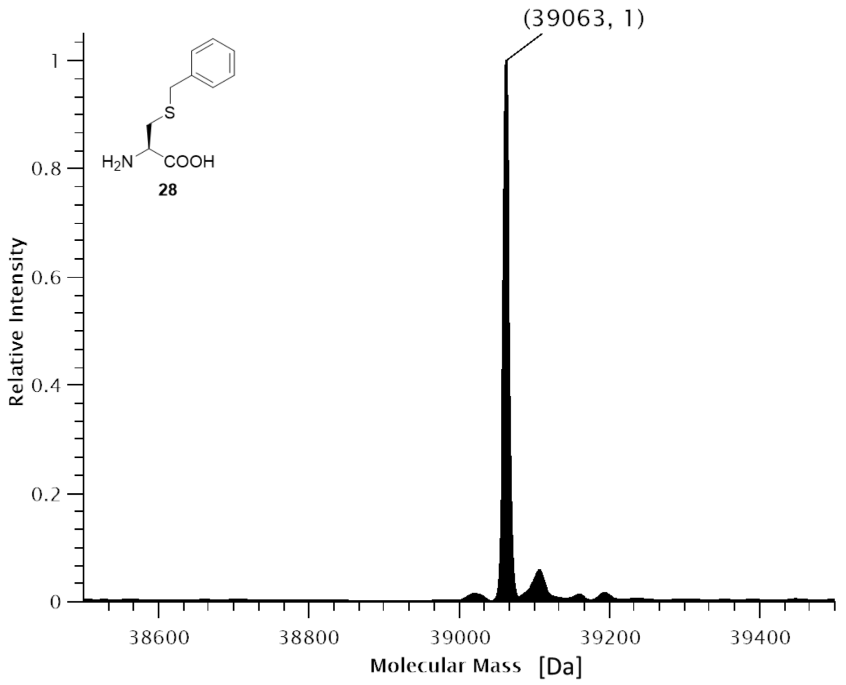

Supplementary Figure S43: Deconvoluted ESI-MS spectrum of SUMO-sfGFP(R2(**ncAA**))-His6 production in *E. coli* BL21(DE3) with co-expression of SmbP-*Mb*SacRS(N311M:C313W:V366A:W382N). Expected protein mass: 39061 Da. Observed mass: 39063 Da.

2.4. DNA sequences used in this study

The following section contains DNA sequences used in this study. For clarity, important regions of constructs are highlighted by color.

1) SmbP-(GGSH-linker)-*Mb*PylRS(T13I:I36V:N311:C313:Y349F:V366:W382:)

ATGAGCGGTCATACCGCACATGTTGATGAAGCAGTTAAACATGCCGAAGAAGCAGTT
GCACACGGTAAAGAAGGCCATACCGATCAGCTGCTGGAACATGCAAAAGAAAGTCTGACC
CATGCCAAAGCAGCCAGCGAAGCCGGTGGTAATACCCATGTTGGTCATGGTATTAAACATC
TGGAAGATGCCATCAAACATGGTGAAGAGGGTCATGTTGGTGTTGCGACCAAACACGCAC
AAGAAGCAATTGAACATCTGCGTGCAAGCGAACATAAAAGCCATGGCGGCTCTCATATGG
ACAAAAAACCGCTGGACGTTCTGATTAGCGCAATTGGTCTGTGGATGAGCCGTACCGGCAC
CCTGCATAAAATCAAACATCATGAAGTTAGCCGCAGCAAAGTCTATATTGAAATGGCATGT
GGTGATCATCTGGTGGTGAATAATAGCCGTAGCTGTCGTACCGCACGTGCATTTCGTCATCA
CAAATATCGTAAAACCTGTAAACGTTGCCGTGTTAGCGACGAAGATATTAACAATTTTCTG
ACCCGTAGCACCGAAAGCAAAAATTCAGTTAAAGTTCGTGTTGTGAGCGCTCCGAAAGTTA
AAAAAGCAATGCCGAAAAGCGTTAGTCGTGCACCGAAACCTCTGGAAAATAGCGTTAGCG
CAAAAGCAAGCACCAATACCAGCCGTAGCGTTCCGAGTCCGGCAAAAAGCACCCCGAATA
GCAGCGTTCCGGCAAGCGCACCGGCACCGAGCCTGACCCGTTCACAGCTGGATCGTGTTGA
AGCACTGCTGAGCCCTGAAGATAAAATCAGCCTGAATATGGCAAAACCGTTTCGTGAACTG
GAACCGGAACTGGTTACCCGTCGTAAAAATGATTTTCAGCGTCTGTATACCAACGATCGCG
AAGATTATCTGGGTAAACTGGAACGTGATATTACCAAATTTTTCGTGGATCGCGGTTTTCTG
GAAATCAAAAGCCCGATTCTGATTCCGGCAGAATATGTTGAACGTATGGGCATTAATAACG
ATACCGAACTGAGCAAACAAATCTTCCGCGTTGATAAAAATCTGTGTCTGCGTCCGATGCT
GGCACCGACCCTGTATAACTATCTGCGCAAACTGGATCGTATTCTGCCTGGTCCGATTAAA
ATCTTTGAAGTTGGTCCGTGCTATCGCAAAGAAAGTGATGGTAAAGAACACCTGGAAGAGT
TTACGATGGTTAACTTTTGTCAGATGGGTAGCGGTTGTACCCGTGAAAATCTGGAAGCACTG
ATTAAAGAGTTTCTGGACTATCTGGAAATTGACTTTGAAATTGTTGGCGATAGCTGCATGGT
TTTTGGTGATACCCTGGATATTATGCATGGTGATCTGGAACTGAGTAGCGCAGTTGTTGGTC
CGGTTAGCCTGGATCGCGAATGGGGTATTGATAAACCGTGGATTGGTGCAGGTTTTGGTCTG
GAACGTCTGCTGAAAGTTATGCACGGCTTTAAAAACATTAAACGTGCAAGCCGTTCCGAGA
GCTATTACAATGGTATTAGCACCAACCTGTAA

2) His6-SUMO-sfGFP(R2amber)-strep

ATGGGCAGCAGCCATCATCATCATCATCACGGTTCTGACTCCGAAGTCAATCAAGAAG
CTAAGCCAGAGGTCAAGCCAGAAGTCAAGCCTGAGACTCACATCAATTTAAAGGTGTCCG
ATGGATCTTCAGAGATCTTCTTCAAGATCAAAAAGACCACTCCTCTGCGTCGTCTGATGGAA
GCGTTCGCTAAAAGACAGGGTAAGGAAATGGACTCCTTAAGATTCTTGTACGACGGTATTA
GAATCCAAGCTGATCAGACCCCTGAAGATTTGGACATGGAGGATAACGATATTATTGAGGC
TCATCGCGAACAGATTGGTGGCATGTAGAAAGGCGAAGAGCTGTTCACTGGTGTCGTCCCT
ATTCTGGTGGAACTGGATGGTGATGTCAACGGTCATAAGTTTTCCGTGCGTGGCGAGGGTG
AAGGTGACGCAACTAATGGTAAACTGACGCTGAAGTTCATCTGTACTACTGGTAAACTGCC
GGTACCTTGGCCGACTCTGGTAACGACGCTGACTTATGGTGTTCAGTGCTTTGCTCGTTATCC
GGACCATATGAAGCAGCATGACTTCTTCAAGTCCGCCATGCCGGAAGGCTATGTGCAGGAA
CGCACGATTTCCTTTAAGGATGACGGCACGTACAAAACGCGTGCGGAAGTGAAATTTGAA
GGCGATACCCTGGTAAACCGCATTGAGCTGAAAGGCATTGACTTTAAAGAAGACGGCAAT
ATCCTGGGCCATAAGCTGGAATACAATTTTAACAGCCACAATGTTTACATCACCGCCGATA
AACAAAAAAATGGCATTAAAGCGAATTTTAAAATTCGCCACAACGTGGAGGATGGCAGCG
TGCAGCTGGCTGATCACTACCAGCAAAACACTCCAATCGGTGATGGTCCTGTTCTGCTGCC
AGACAATCACTATCTGAGCACGCAAAGCGTTCTGTCTAAAGATCCGAACGAGAAACGCGA

TCATATGGTTCTGCTGGAGTTCGTAACCGCAGCGGGCATCACGCATGGTATGGATGAACTG
TACAAAAGCGCTTGGAGCCACCCGCAGTTCGAAAAATAA

3) SUMO-sfGFP(R2amber)-His6

ATGGGCAGCAGCGACTCCGAAGTCAATCAAGAAGCTAAGCCAGAGGTCAAGCCAGA
AGTCAAGCCTGAGACTCACATCAATTTAAAGGTGTCCGATGGATCTTCAGAGATCTTCTTCA
AGATCAAAAAGACCACTCCTCTGCGTCGTCTGATGGAAGCGTTCGCTAAAAGACAGGGTA
AGGAAATGGACTCCTTAAGATTCTTGTACGACGGTATTAGAATCCAAGCTGATCAGACCCC
TGAAGATTTGGACATGGAGGATAACGATATTATTGAGGCTCATCGCGAACAGATTGGTGGC
ATGTAGAAAGGCGAAGAGCTGTTCACTGGTGTCGTCCCTATTCTGGTGGAACTGGATGGTG
ATGTCAACGGTCATAAGTTTTCCGTGCGTGGCGAGGGTGAAGGTGACGCAACTAATGGTAA
ACTGACGCTGAAGTTCATCTGTACTACTGGTAAACTGCCGGTACCTTGGCCGACTCTGGTAA
CGACGCTGACTTATGGTGTTCAGTGCTTTGCTCGTTATCCGGACCATATGAAGCAGCATGAC
TTCTTCAAGTCCGCCATGCCGGAAGGCTATGTGCAGGAACGCACGATTTCCTTTAAGGATG
ACGGCACGTACAAAACGCGTGCGGAAGTGAAATTTGAAGGCGATACCCTGGTAAACCGCA
TTGAGCTGAAAGGCATTGACTTTAAAGAAGACGGCAATATCCTGGGCCATAAGCTGGAATA
CAATTTTAACAGCCACAATGTTTACATCACCGCCGATAAACAAAAAAATGGCATTAAAGC
GAATTTTAAAATTCGCCACAACGTGGAGGATGGCAGCGTGCAGCTGGCTGATCACTACCAG
CAAAACACTCCAATCGGTGATGGTCCTGTTCTGCTGCCAGACAATCACTATCTGAGCACGC
AAAGCGTTCTGTCTAAAGATCCGAACGAGAAACGCGATCATATGGTTCTGCTGGAGTTCGT
AACCGCAGCGGGCATCACGCATGGTATGGATGAACTGTACAAAAGCGCTCATCATCATCAT
CATCACTAA

References:

31. Owens, A.E.; Grasso, K.T.; Ziegler, C.A.; Fasan, R. Two-Tier Screening Platform for Directed Evolution of Aminoacyl–tRNA Synthetases with Enhanced Stop Codon Suppression Efficiency. *ChemBioChem* **2017**, *18*, 1109–1116, doi:10.1002/cbic.201700039.
